# Identification by mass spectrometry and immunoblotting of xenogeneic antigens in the *N*- and *O*-glycomes of porcine, bovine and equine heart tissues

**DOI:** 10.1007/s10719-020-09931-1

**Published:** 2020-06-15

**Authors:** Chunsheng Jin, Reeja Maria Cherian, Jining Liu, Heribert Playà-Albinyana, Cesare Galli, Niclas G. Karlsson, Michael E. Breimer, Jan Holgersson

**Affiliations:** 1 Department of Medical Biochemistry, Institute of Biomedicine Sahlgrenska Academy, University of Gothenburg, Gothenburg, Sweden; 2 Department of Surgery, Institute of Clinical Sciences, Sahlgrenska Academy at University of Gothenburg, Göteborg, Sweden; 3 Department of Clinical Chemistry and Transfusion Medicine, Institute of Biomedicine, Sahlgrenska Academy, University of Gothenburg, Gothenburg, Sweden; 4 Department of Biochemistry and Biotechnology, Faculty of Chemistry, Rovira i Virgili University, Tarragona, Spain; 5 Avantea Laboratory of Reproductive Technologies, Cremona, Italy; 6 Avantea Foundation, Cremona, Italy

**Keywords:** Bioprosthetic heart valves, structural valve deterioration, glycome, xenogeneic antigen, liquid chromatography – tandem mass spectrometry

## Abstract

Animal bioprosthetic heart valves (BHV) are used to replace defective valves in patients with valvular heart disease. Especially young BHV recipients may experience a structural valve deterioration caused by an immune reaction in which α-Gal and Neu5Gc are potential target antigens. The expression of these and other carbohydrate antigens in animal tissues used for production of BHV was explored. Protein lysates of porcine aortic and pulmonary valves, and porcine, bovine and equine pericardia were analyzed by Western blotting using anti-carbohydrate antibodies and lectins. *N*-glycans were released by PNGase F digestion and *O*-glycans by β-elimination. Released oligosaccharides were analyzed by liquid chromatography – tandem mass spectrometry. In total, 102 *N*-glycans and 40 *O*-glycans were identified in animal heart tissue lysates. The *N*- and *O*-glycan patterns were different between species. α-Gal and Neu5Gc were identified on both *N*- and *O*-linked glycans, *N,N´*-diacetyllactosamine (LacdiNAc) on *N*-glycans only and sulfated *O*-glycans. The relative amounts of α-Gal-containing *N*-glycans were higher in bovine compared to equine and porcine pericardia. In contrast to the restricted number of proteins carrying α-Gal and LacdiNAc, the distribution of proteins carrying Neu5Gc-determinants varied between species and between different tissues of the same species. Porcine pericardium carried the highest level of Neu5Gc-sialylated *O*-glycans, and bovine pericardium the highest level of Neu5Gc-sialylated *N*-glycans. The identified *N-* and *O-*linked glycans, some of which may be immunogenic and remain in BHVs manufactured for clinical use, could direct future genetic engineering to prevent glycan expression rendering the donor tissues less immunogenic in humans.

**Electronic supplementary material:**

The online version of this article (10.1007/s10719-020-09931-1) contains supplementary material, which is available to authorized users.

## Introduction

More than 250,000 heart valves are replaced worldwide each year due to valvular heart disease [1]. Approximately 55% of the valves are mechanical (MHV) and 45% are bioprosthetic heart valves (BHV) [1]. MHV have long-term durability, but patients with MHV require lifelong anticoagulation and suffer from thereto associated risks of spontaneous bleedings, thrombosis and thromboembolism [1, 2]. Whenever possible a BHV is the preferred valve type because patients receiving BHV do not need long-term anticoagulation. BHV are manufactured from porcine valvular tissue or porcine, bovine or equine pericardial tissue, which has been treated in a process that includes glutaraldehyde treatment [1–3]. BHV are susceptible to structural valve deterioration (SVD), which is characterized by valve thickening and calcification [4, 5] and occurs in >10% of implanted BHV within 10 years [2] and can therefore not be used in the younger patient cohort. The exact mechanism behind this degeneration process is not known, but it is believed to be due to a chemical process between the glutaraldehyde preservative and free calcium ions present in the blood. In addition, an immune response to the xenogeneic BHV tissue may also contribute as it is mostly seen in young patients with a robust immune system [1, 2, 6].

Although the immune response has been suggested to contribute to SVD, devitalization of cells in the tissue renders the response weaker than what is seen following exposure to living xenogeneic tissue after which hyperacute or acute rejection ensues [3]. Residual cells or cell remnants devitalized by glutaraldehyde treatment may initiate calcification and SVD following binding of immunoglobulins to the valve matrix, complement activation and subsequent recruitment of macrophages [7, 8]. Cell surface glycans linked to proteins or lipids may act as targets for natural preformed antibodies [9–11]. The Galα1,3Galβ1,4GlcNAc-R (α-Gal) determinant expressed on porcine, bovine and equine tissues is the predominant glycoantigen for antibodies mediating hyperacute rejection of vascularized porcine xenografts in humans and non-human primates [12]. The α-Gal determinant has been demonstrated on fibrocytes interspersed in the connective tissue of fixed and native porcine valves [13, 14] and patients receiving porcine BHV exhibited a rise in cytotoxic anti-Gal IgM antibodies [13]. A direct role of anti-Gal antibodies in the calcification process has also been suggested [15]. In a recent study by W. Lee et al, α-Gal was detected on the cells and connective tissue of fresh and glutaraldehyde-fixed porcine heart valve as well as pericardial tissue [8]. Using immunohistochemical techniques the authors did not find any significant difference in α-Gal expression between porcine valve tissue (proximal, middle and distal parts) and pericardium [8]. Other potential xenoantigens that may contribute to SVD include glycans capped by the *N*-glycolylneuraminic acid (Neu5Gc), which is lacking in humans because of a deletion in the *CMAH* gene encoding the CMP-Neu5Ac hydroxylase [16]. Neu5Gc is expressed on native pig heart valves and pericardium [8, 17, 18], including six commercial valve types [19]. A detailed analysis of porcine, bovine and equine pericardia glycolipids revealed several potentially immunogenic carbohydrate determinants such as α-Gal, blood group A, Forssman and Neu5Gc [20]. Interestingly, Neu5Gc-terminated glycosphingolipids could not be detected in the ganglioside (acidic glycosphingolipids) fractions isolated from native porcine aortic and pulmonary valve cusps [21]. Other non-α-Gal glycan determinants which humans may have naturally occurring antibodies against include, but are not limited to, Galβ1,3GalNAcα1-R (Thomsen-Friedenreich antigen), Sid blood group (Sd^a^)-like antigens, terminal α-linked GalNAc, β3-linked Gal, sulfatide and the blood group pk antigen [22].

This study expands our knowledge regarding *N*- and *O*-glycan structures in animal tissues utilized to produce BHV used in the clinic. Western blotting and liquid chromatography – tandem mass spectrometry was used to investigate the *N*- and *O*-glycomes in lysates of native porcine aortic and pulmonary valves, and porcine, bovine and equine pericardia to determine the representation of protein-linked glycans focusing on defining the core chains carrying α-Gal and Neu5Gc xenogeneic determinants, as well as potentially new xenogeneic carbohydrate determinants.

## Experimental Procedures

### Experimental design and statistical rationale

Five different animal heart tissues were characterized regarding their *N*- and *O*-glycomes: pulmonary and aortic valve tissue from porcine hearts (*n*=15 animals), and porcine (*n*=3), bovine (n=3) and equine (n=1) pericardia. Animal tissues were obtained from the slaughterhouse and transported in plastic bags on ice to the laboratory. Selected tissues/valves were excised upon arrival, rinsed four times with phosphate-buffered saline (PBS), and stored at -80°C. Primary human aortic endothelial cells (HAECs; Cascade Biologics, Portland, OR, U.S.A.), known to lack the α-Gal and Neu5Gc determinants, were included for comparison. HAECs at passages 10-15 were cultured in endothelial cell growth medium (EBM-2; Lonza Group Ltd, Basel, Switzerland) supplemented with 20% fetal bovine serum (FBS; ThermoFischer Scientific, MA, U.S.A). Cells were maintained in a humidified incubator at 37°*C* and 5.0% CO_2_.

### Total protein extraction and quantification

Tissues from multiple animals where pooled and homogenized with a polytron in 1:20 (*w*/*v*) of tissue to T-PER (Tissue-Protein Extract Reagent), pH 7.6 (ThermoFischer Scientific), in the presence of a protease inhibitor cocktail that inhibits serine, cysteine, aspartic and metalloproteases (Sigma-Aldrich, St Louis, MO, U.S.A). According to manufacturer instructions, tissue debris was pelleted at 10,000g for 5 minutes at 4°C and the lysate collected. For HAEC protein extraction, cells in culture flasks were washed three times in PBS and removed by scraping. Cells were collected by centrifugation at 200g for 5 minutes. T-PER was added to the cell pellet and was gently shaken for 10 minutes. The cell debris was removed by centrifugation 10,000g for 10 minutes at 4°C and the lysate collected. Protein concentrations were determined using the BCA protein assay kit (Pierce^TM^, ThermoFischer Scientific) according to the manufacturer’s instructions. Protein lysates were stored frozen at -80oC until analyzed.

### Antibodies, lectins, neoglycoproteins and recombinant mucin-type fusion proteins

Affinity-purified chicken IgY anti-Neu5Gc (BioLegend, San Diego, CA, USA), human anti-α-Gal antibodies (mainly IgG and IgM) purified from human AB serum (Sigma-Aldrich) by using an affinity matrix functionalized with Galα1,3Galβ1,4GlcNAcβ1,3Galβ1,4Glc (ELICITYL, Crolles, France), mouse anti-LacdiNAc (anti-LDN, IgM, clone SMLDN1.1; generously provided by Prof Richard D Cummings, Harvard University), mouse anti-Sd^a^ (IgM, KM694; Tokyo Research Laboratories, Tokyo, Japan), mouse anti-Lewis^a^ (Le^a^; IgG1, clone KM231; Calbiochem, San Diego, CA, USA), mouse anti-Lewis^b^ (Le^b^; IgM, T218; Santa Cruz Biotechnology, Santa Cruz, CA, USA), mouse anti-sialyl-Lewis^x^ (sLe^x^; anti-CD15s, IgM, CSLEX1; BD PharMingen, San Diego, CA, USA), mouse anti-Le^x^ (CD15 antibody; IgM; Santa Cruz Biotechnology), and mouse anti-Lewis^y^ (Le^y^; IgM F3; ThermoFischer Scientific) were used as primary antibodies. Peroxidase-conjugated donkey anti-chicken IgY (Jackson ImmunoResearch, Westgrove, PA, USA), goat anti-human IgG (Sigma-Aldrich), goat anti-human IgM (Sigma-Aldrich), goat anti-mouse IgM (Sigma-Aldrich), and goat anti-mouse IgG F(ab)’_2_ (Sigma-Aldrich) were used as secondary antibodies.

Biotinylated *Maackia amurensis* lectin (MAL-1 and MAL-2) and *Sambucus nigra* bark lectin (SNA) were from Vector laboratories (Burlingame, CA, U.S.A.) as was the peroxidase-conjugated avidin D.

Purified recombinant mucin-type fusion protein, CP-55, produced in CHO-K1 cells stably transfected with the PSGL-1/mIgG2b plasmid and carrying mono- and disialylated core 1 *O*-glycans [23] was used as a negative control (Table 1) for the Western blotting. Le^a^-BSA, Le^b^-BSA and sLe^x^-BSA (Dextra Laboratories, Reading, UK) were used as positive controls for the respective Lewis antibody staining. For other antibody and lectin stainings, recombinant mucins decorated with different glycan determinants and described in previous publications, were used as positive controls. They included C-PGC2, carrying terminal α-Gal [24, 25]; CP-C3, predominantly substituted with core 3 *O*-glycans carrying the type 2 chain (Galβ1,4GlcNAc) [26]; CP-ext C1 decorated with extended core 1 *O*-glycans terminated with α2,3-linked sialic acid [27]; and CP-ext C1-ST6 carrying extended core 1 *O*-glycans terminated with α2,6-linked sialic acid [28]. CP-LDN carrying the LacdiNAc determinant on core 2 *O*-glycans of PSGL-1/mIgG_2b_ was produced by co-expressing the human *B4GALNT3* encoding β1,4-*N*-acetylgalactosaminyltransferase 3, *GCNT1* encoding β1,6-*N*-acetylglucosaminyltransferase 1 and PSGL-1/mIgG2b cDNA in CHO-K1 cells (unpublished result). The CP-Neu5Gc fusion protein expressing a higher ratio of terminal Neu5Gc to Neu5Ac on PSGL-1/mIgG2b was produced by culturing CHO-K1 cells stably transfected with PSGL-1/mIgG2b, human β1,3-*N*-acetylglucosaminyltransferase 3 (B3GNT3) and β-galactoside α-2,6-sialyltransferase 1 (ST6GAL1) cDNAs in Neu5Gc-supplemented medium (unpublished result). CP-Le^x^, carrying Lewis x determinants, was produced in CHO-K1 cells by co-expressing the human α1,3-fucosyltransferase 4 (FUT4), the B3GNT3, and the PSGL-1/mIgG2b cDNA. CP-Le^y^, carrying Lewis Y determinants, was produced by co-expressing PSGL-1/mIgG2b, human B3GNT3, the human α1,2-fucosyltransferase 1 (FUT1) and the α1,3/4-fucosyltransferase 3 (FUT3) in CHO-K1 cells [26].

**Table 1:** Antibodies and lectins used for Western blot experiments

Primary antibodies or lectins	Secondary antibodies or lectins	Specificity	Negative control	Positive control
Chicken anti-Neu5Gc IgY (1:2,000)	Peroxidase-conjugated donkey anti-chicken IgY (1:50,000)	Neu5Gc	C-P55	CP-NeuGc
Human anti-αGal IgG+IgM (1.68 mg/ml)	Peroxidase-conjugated goat anti-human IgG (1:20,000) Peroxidase-conjugated goat anti-human IgM (1:10,000)	αGal	none	C-PGC2
Anti-LDN IgM SMLDN1.1 (1:50)	Peroxidase-conjugated goat anti-mouse IgM (1:20,000)	LacdiNAc	C-P55	CP-LDN
KM694 (5 μg/ml)	Peroxidase-conjugated goat anti-mouse IgM (1:20,000)	Sd^a^	C-P55	none
Anti-Lewis a (1:500)	peroxidase-conjugated goat anti-mouse IgG Fab (1:10,000)	Lewis a	C-P55	Le^a^-BSA
Anti-Lewis b (1:1,000)	Peroxidase-conjugated goat anti-mouse IgM (1:10,000)	Lewis b	C-P55	Le^b^-BSA
Anti-Lewis x (1:800)	Peroxidase-conjugated goat anti-mouse IgM (1:10,000)	Lewis x	C-P55	CP-Le^x^
Mouse anti-human CD15s (1:1,000)	Peroxidase-conjugated goat anti-mouse IgM (1:10,000)	sialyl-Lewis x	C-P55	sLe^x^-BSA
Blood group Lewis y F3 (1:500)	Peroxidase-conjugated goat anti-mouse IgM (1:10,000)	Lewis y	C-P55	CP-Lewis Y
MAL-1 (1 μg/ml)	Peroxidase-conjugated avidin D (1 μg/ml)	(α2,3-Neu5Ac/Gc)Galβ4GlcNAc	none	CP-C3
MAL-2 (1 μg/ml)	Peroxidase-conjugated avidin D (1 μg/ml)	α2,3-sialic acids	none	CP-ext C1
SNA (1 μg/ml)	Peroxidase-conjugated avidin D (1 μg/ml)	α2,6-sialic acids	none	CP-ext C1-ST6

### SDS-PAGE and Western blotting

Total protein lysates from heart valve and pericardial tissues were dissolved in 2 × lithium dodecyl sulfate (LDS) sample buffer (ThermoFischer Scientific) and incubated at 70°C for 10 minutes. SDS-PAGE was done under non-reducing conditions using 3-8% Tris-acetate gradient gels and Tris-acetate SDS running buffer (ThermoFischer Scientific). Proteins were visualized using SYPRO® Ruby protein gel stain (ThermoFischer Scientific). To detect glycosylated proteins, the SDS-PAGE protein gels were stained using the Pro Q Emerald 300 glycoprotein detection kit (ThermoFischer Scientific). The Candycane precision standard (ThermoFischer Scientific) was applied as a reference for protein molecular weight determination in the Ruby and Pro-Q gels. For antibody and lectin staining, precision protein standard (Hi-Mark^TM^, ThermoFischer Scientific) was applied as reference for protein molecular weight determination. These gels were visualized in an imaging system (MF-ChemiBIS 2.0, DNR Bio-Imaging Systems Ltd, Jerusalem, Israel).

For Western blotting, separated proteins were electrophoretically blotted onto nitrocellulose membranes (ThermoFischer Scientific) using an iBlot (ThermoFischer Scientific). For antibody staining (except in the case of anti-Neu5Gc Ab staining), membranes were blocked with 3% BSA in PBS with 0.2% Tween 20 (PBS-T). For Neu5Gc staining, membranes were blocked with 0.5% gelatin from cold water fish skin (Sigma-Aldrich) in PBS-T. For lectin staining, membranes were blocked with Carbo-Free Blocking solution (Vector laboratories, Burlingame, CA, USA) for 1 hr. The membranes were then incubated at room temperature for 2 hours with primary antibodies or biotinylated lectins (Table 1) diluted in PBS-T. After washing with PBS-T, membranes were incubated for 1 hour at room temperature with peroxidase-conjugated secondary antibodies or avidin (Table 1) diluted in PBS-T. After each incubation, membranes were washed five times with PBS-T. Bound antibodies and lectins were visualized by chemiluminescence using the ECL kit according to the manufacturer’s instructions (GE Healthcare, Uppsala, Sweden).

### Release of N- and O-glycans from solubilized proteins

The tissue extracts (100 μl) of porcine valve tissues (pulmonary 3 μg/ml, aortic 2.4 μg/ml), pericardia (porcine 2.9 μg/ml, bovine 1.6 μg/ml and equine 2.6 μg/ml), and HAECs (1 μg/ml) were diluted in 7 M urea to a final volume of 200 μl. The release of glycans was performed as described previously with a few modifications [29]. The protease inhibitors included in the lysate buffer were removed by spinning through a 30 kDa spin column (Millipore, Bedford, MA, USA) at 11,000 rpm for 5 min. About 60 μl of sample was incubated with 25 mM DTT and sequencing grade trypsin (1% *w*/*w*; Promega, Nacka, Sweden) at 37°C overnight. Tryptic peptides were precipitated with 80% (*v*/*v*) acetone. The dried pellet was washed with cold 50% methanol. The dried pellet was incubated over night at 37°C with 5 units of PNGase F (Prozyme, Hayward, CA, USA) in 50 mM NH_4_HCO_3_, pH 8.4.

Released *N*-glycans were separated from peptides on a Sep-Pak C18 cartridge (Waters, Milford, MA), pre-washed with 100% methanol. After loading the sample, the C18 cartridge was rinsed with 0.1% trifluoroacetic acid (TFA). The flow-through and wash fractions contained released *N*-glycans. Peptides and *O*-glycopeptides were eluted with 80% acetonitrile containing 0.1% TFA. Fractions containing *N*-glycans and peptides/*O*-glycopeptides, respectively, were dried in a SpeedVac. Released *N*-glycans were reduced by 0.5 M NaBH_4_ in 10 mM NaOH at 50°C over night. *O*-glycans were released by reductive β-elimination using 50 M NaBH_4_ in 50 mM NaOH at 50°C over night. Reactions were quenched with glacial acetic acid, and samples were desalted and dried as previously described [30].

Released glycans were analyzed by LC-MS using an in-house prepared, 10 cm × 150 μm I.D. column containing 5 μm porous graphitized carbon particles (Thermo Scientific, Waltham, MA, USA). Glycans were eluted using a linear gradient of 0–40% acetonitrile in 10 mM NH_4_HCO_3_ over 40 min at a flow rate of 10 μl/min. Eluted glycans were detected using a LTQ ion trap mass spectrometer (Thermo Scientific) in negative ion mode with an electrospray voltage of 3.5 kV, a capillary voltage of -33.0 V and a capillary temperature of 300°C. Air was used as sheath gas and mass ranges were defined dependent on the specific structure to be analyzed. The data were processed using the Xcalibur software (version 2.0.7, Thermo Scientific). Glycans were identified from their MS/MS spectra by manual annotation. For structural annotation, the biosynthesis of *N*- and *O*-glycans was assumed to follow the classical pathways. Diagnostic fragmentation ions for *N*- and *O*-glycans were investigated as described [31]. Terminal Hex_2_ units were presumed to be αGal and terminal HexNAc_2_ determinants were presumed to be LacdiNAc. Chain elongation was expected to be mediated by the addition of *N*-acetyllactosamine units. The annotated structures are submitted to the UniCarb-DB database (http://unicarb-dr.biomedicine.gu.se/references/343) and will be included in the next release.

For comparison of glycan abundances between samples, individual glycan structures were quantified relative to the total content by integration of the extracted ion chromatogram peak area. The area under the curve (AUC) of each structure was normalized to the total AUC and expressed as a percentage.

## Results

### Protein expression patterns and total N- and O-glycan repertoires in animal heart tissues

**Fig. 1:**
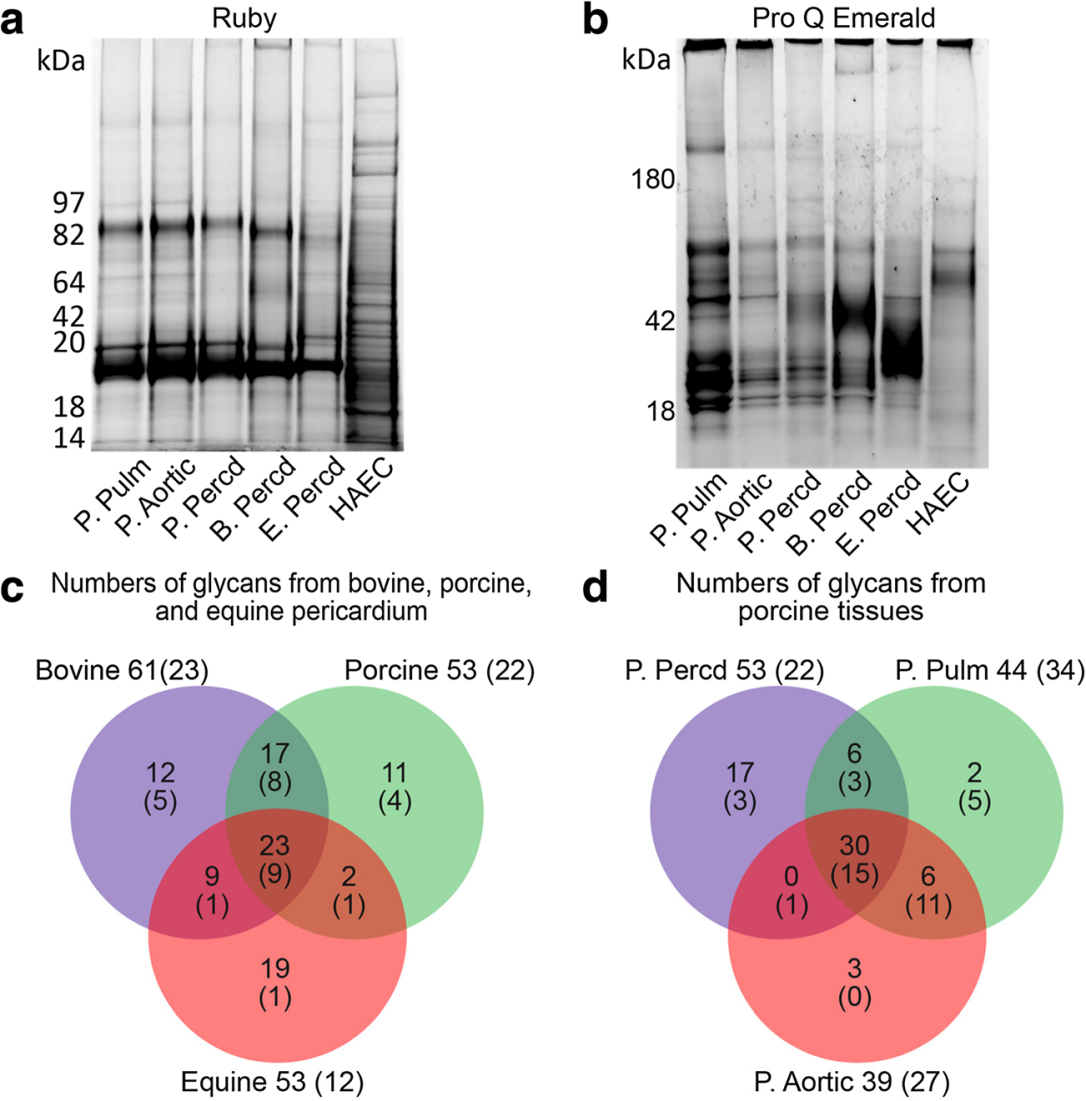
SDS-PAGE analysis of protein extracts from porcine pulmonary (P. Pulm) and aortic (P. Aortic) heart valve tissue, porcine (P. Percd), bovine (B. Percd) and equine (E. Percd) pericardium. A human aortic endothelial cell lysate was used as negative control. Gels were stained with Ruby (**a**) and Pro Q Emerald (**b**). *N*- and *O*-glycans from these samples were characterized by PGC LC-MS/MS. The number of characterized *N*- (no brackets) and *O*-glycans (brackets) of different species (**c**) and different porcine tissues (**d**) are shown

Proteins solubilized from porcine, bovine and equine heart tissues were detected by SYPRO Ruby A (Fig. 1a). Proteins solubilized from human aortic endothelial cells were stained for comparison as negative control. Two major protein components with an apparent molecular weight of between 82 and 97 kDa and approximately 19 kDa were detected in all porcine tissues. The latter was seen also in bovine and equine pericardium, while a component of similar size to the former was detected in bovine and weakly in equine pericardium. These components were not detected in the human aortic endothelial cellprotein lysate, which exhibited a distinctively different Ruby staining pattern from the animal tissues. Detection of glycosylated proteins by the Pro Q Emerald staining (Fig. 1b), revealed a less consistent staining pattern between the different tissues (compare porcine valve tissue and pericardium) and between tissues of different species (compare the pericardial tissues of different species). The Pro Q Emerald staining of the porcine pulmonary and aortic valve tissues appeared similar even though the pulmonary valve lysate stained stronger than the aortic valve lysate (Fig. 1b).

In total, LC-MS/MS analysis revealed 102 *N*-glycans, 40 *O*-glycans and 2 linker regions of proteoglycans in the protein lysates of porcine (valves and pericardium), bovine and equine heart tissues combined (Table S1). Bovine pericardium displayed the highest number of *N*- and *O*-glycans (61 and 23), followed by the porcine (53 and 22) and equine (53 and 12) pericardia. Only 23 *N*-glycans out of the total 93 and 9 *O*-glycans out of the total 29 were detected in all species (Fig. 1c). This suggests that most *N*- and *O*-glycans from porcine, equine and bovine heart tissues are structurally different. There was also structural glycan diversity between the different porcine tissues (Fig. 1d); only 30 out of 64 *N*-glycans and 15 out of 38 *O*-glycans were detected in all porcine heart tissues. *N*- and *O*-glycan structures common between species were predominant, but tissue-specific structures were also detected. *N*- and *O*-glycans carrying established and putative xenogeneic carbohydrate determinants are characterized in detail below.

### Distribution of the α-Gal determinant

**Fig. 2:**
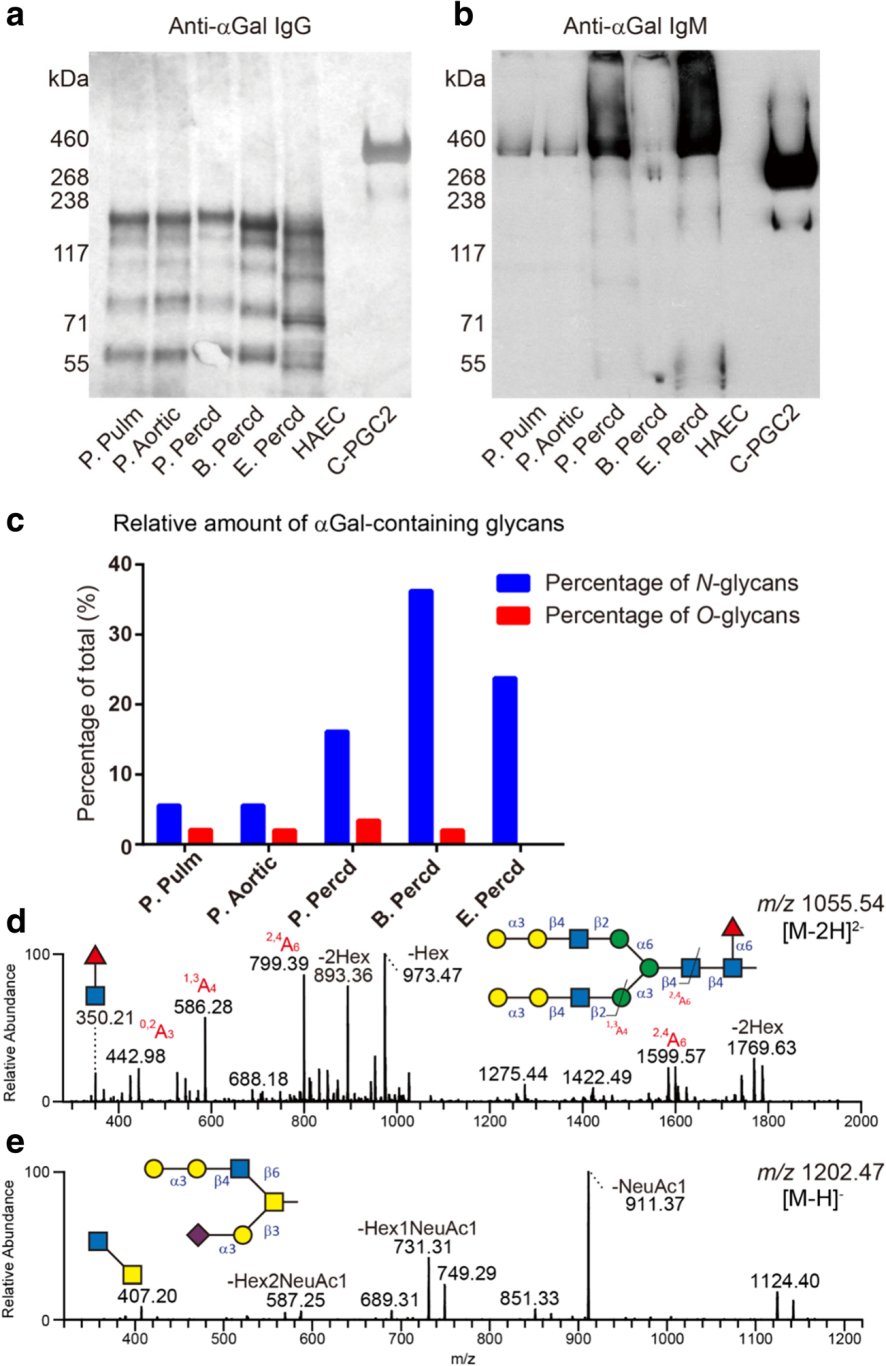
Western blot analysis of protein extracts from animal heart valves and pericardia using affinity-purified human anti-α-Gal IgG (**a**) and IgM (**b**). A human aortic endothelial cell lysate was used as negative control. The positive control was a recombinant mucin-type fusion protein (C-PGC2) carrying terminal α-Gal residues. The relative amounts of individual structures are given in percentage (%) of the total sum of integrated peak areas in the LC-MS chromatograms. Relative amounts of α-Gal-containing *N*- and *O*-glycans in these samples are shown (**c**). MS/MS spectra of the predominant α-Gal-containing *N*- and *O*-glycan, respectively, with masses corresponding to structures having the following saccharide compositions: Hex_7_HexNAc_4_dHex_1_ ([M-2H]^2-^ of *m/z* 1055) and Neu5Ac_1_Hex_3_HexNAc_2_ ([M-H]^-^ of *m/z* 1202), are shown in **d** and **e**, respectively

The presence of α-Gal determinants on proteins solubilized from the tissues was assessed by Western blot using anti-α-Gal antibodies purified from human AB serum. Even though anti-α-Gal IgG (Fig. 2a) and IgM (Fig. 2b) exhibited distinctly different binding patterns based on molecular weight, α-Gal staining was similar between species and between different porcine heart tissues (Fig. 2a and b). As expected, anti-α-Gal antibodies did not react with the human aortic endothelial cellprotein lysate (Fig. 2a and b).

In line with the Western blot results, terminal Hex-Hex sequences (assumed to be α-Gal-containing glycans) were detected by LC-MS/MS in the porcine, bovine and equine pericardia. In total, α-Gal terminals were identified in 18 *N*-glycans and 6 *O*-glycans (Table S1). The relative amount (as determined by measuring the area under the curve for α-Gal containing glycans divided by the total area under the curve) was used to display differences of α-Gal containing *N*-glycans, acknowledging the fact that the low number of animals prevented statistical comparison. In bovine pericardium 36% of the intensity constituted tentative α-Gal *N*-glycans and was found to be higher compared to equine (24%) and porcine (16%) pericardia (Fig. 2c and Table 2). In all the samples, the relative amounts of α-Gal containing *O*-glycans were low (2-3%, Fig. 2c and Table 2) compared to *N*-glycans. *O*-glycans containing α-Gal structures were not detected on equine pericardium proteins.

**Table 2:** The relative amount of αGal-containing, sialylated and sulfated glycans identified in porcine, bovine and equine tissues

Species	Tissues/Cells	αGal (%)		Neu5Gc (%)		Neu5Ac (%)		Sulfate (%)
		*N*-glycan	*O*-glycan	*N*-glycan	*O*-glycan	*N*-glycan	*O*-glycan	*O*-glycan
Bovine	Percd	36	2	42	16	45	78	12
Equine	Percd	24	ND	14	15	65	67	11
Porcine	Percd	16	3	13	65	67	44	9
	Pulm valve	6	2	8	8	83	85	41
	Aortic valve	6	2	12	7	82	94	32

A difference in the number of structures and amounts of α-Gal containing *N-*glycans was observed in porcine tissues with a more diverse repertoire detected in the pericardium. This is consistent with the findings from another investigation of porcine heart tissue [32]. The dominant α-Gal containing family of *N-*glycans in all the tissues were core α1,6-fucosylated bi-antennary with or without terminal sialylation. This type of structures is exemplified in Fig. 2d by an *N*-glycan structure with two terminal α-Gal residues ([M-2H]^2-^ ion of *m/z* 1055.54, Fig. 2d and Table S1). The majority of α-Gal containing *O-*glycans had the determinant on the C6 branch of core 2 (i.e., Galβ1,3(Galα1,3Galβ1,4GlcNAcβ1,6)GalNAcol; Fig. 2e and Table S1).

### Distribution of Neu5Gc determinants

**Fig. 3:**
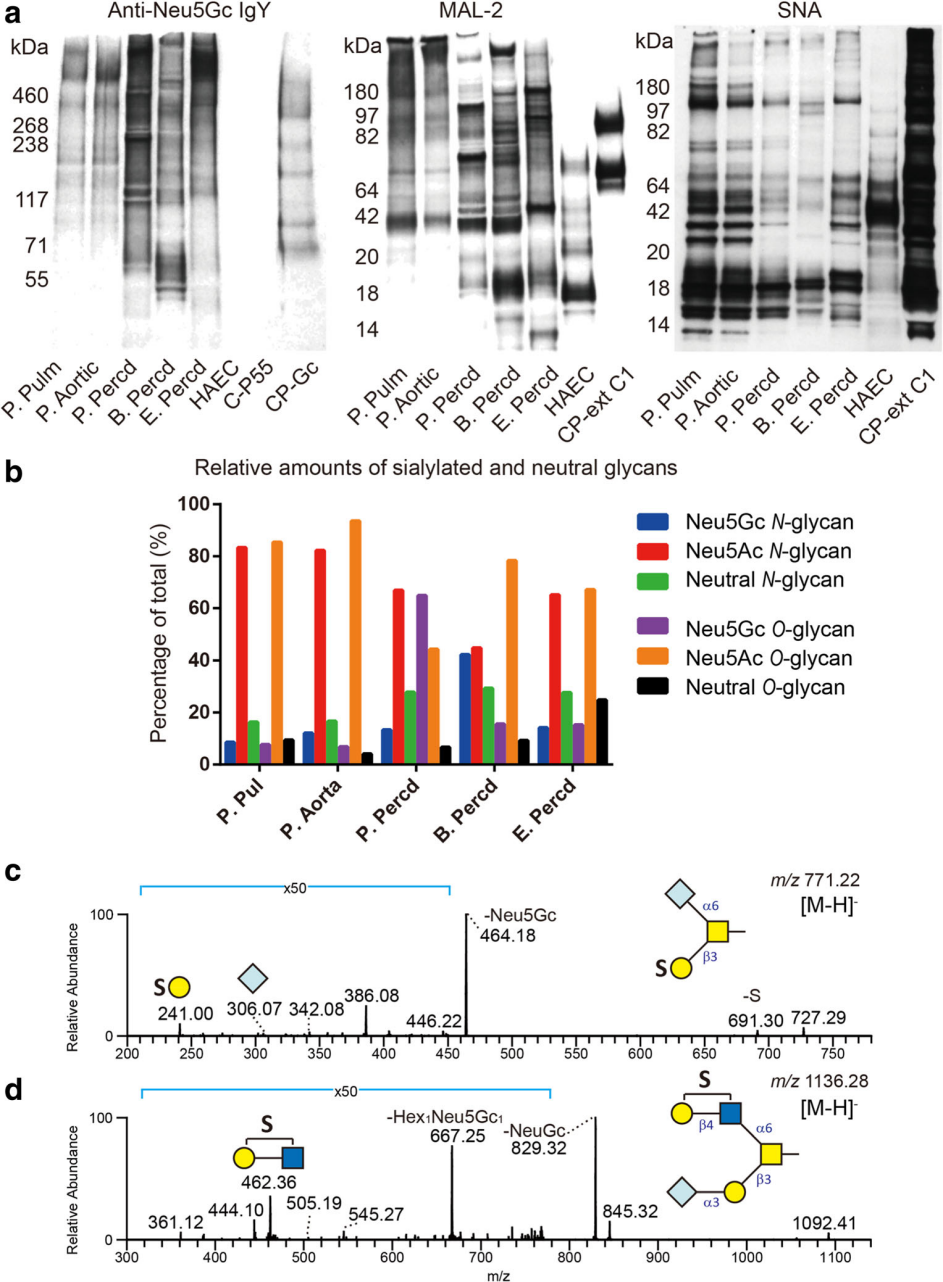
Western blot analysis of protein extracts from animal heart valves and pericardia using chicken IgY anti-Neu5Gc, and MAL-2 and SNA lectins (**a**). Recombinant mucin-type fusion proteins – CP-Gc carrying Neu5Gc structures, CP-ext C1 carrying extended core 1 *O*-glycans terminated with α2,3-linked sialic acid and CP-ext C1-ST6 carrying extended core 1 *O*-glycans terminated with α2,6-linked sialic acid – were used as positive controls for anti-Neu5Gc, MAL-2 and SNA staining, respectively. The C-P55 fusion protein lacking Neu5Gc was used as negative control for Neu5Gc staining. A human aortic endothelial cell lysate was used as negative control in all panels. Relative amounts of individual structures are given in percentage (%) of the total sum of integrated peak areas in the LC-MS chromatograms. Relative amounts of Neu5Gc-containing *N*- and *O*-glycans in the animal tissue samples are shown in (**b**). MS/MS spectra of two sulfated Neu5Gc-containing *O*-glycans with masses corresponding to a Neu5Gc_1_Hex_1_HexNAc_1_Sul_1_ ([M-H]^-^ of *m/z* 771) and a Neu5Gc_1_Hex_2_HexNAc_2_Sul_1_ ([M-H]^-^ of *m/z* 1136) structure are shown in **c** and **d**, respectively

Western blot analyses of the animal heart tissue protein lysates using anti-Neu5Gc Abs revealed positive staining of several proteins from all species (Fig. 3a). In contrast to the staining pattern of α-Gal-containing proteins that was similar for all species analyzed, staining of proteins carrying Neu5Gc-determinants varied between species and between tissues of the same species (Fig. 3a). A large variation of the mass distribution of proteins between samples was also seen following MAL-2 (α2,3-linked sialic acid) staining, while staining with SNA (α2,6-linked sialic acid) showed less mass variability (Fig. 3a). The lectin staining patterns of porcine aortic and pulmonary valve tissues were very similar, while a distinct difference was found for the valve and pericardial tissues and for the pericardia of different species (Fig. 3a).

Regarding sialic acid-terminating glycans, LC-MS/MS analysis revealed Neu5Gc-containing glycans in all animal tissues (Fig. 3b-d, Table 2 and S1) consistent with the Western blot results. Neu5Ac-containing saccharides dominated (39 *N*-glycans and 17 *O*-glycans), while 32 Neu5Gc-containing glycans were identified (19 *N*-glycans and 13 *O*-glycans). Unlike the relatively high proportion (67% or 12 out of 18) of α-Gal-containing *N*-glycans common between the three species, only 21% (4 out of 19) of the Neu5Gc-containing *N*-glycans and 5% (2 out of 39) of the Neu5Ac-containing *N*-glycans were common for all three species indicating a larger heterogeneity among the sialylated *N*-glycans. The proportion of common sialylated *O*-glycans in all three species was higher, 31% (4 out of 13) for Neu5Gc-containing and 24% (4 out of 17) for Neu5Ac-containing *O*-glycans.

The sialylation level in the animal tissues differed considerably. High levels of *O*-glycan Neu5Ac-sialylation were found in porcine aortic (94%) and pulmonary (85%) valve tissue (Table 2). Porcine pericardium on the other hand showed the lowest degree of Neu5Ac-sialylation of *O*-glycans (44%), but the highest level of Neu5Gc-sialylation (65%) compared to the relative amounts of Neu5Ac- and Neu5Gc-containing *O*-glycans in bovine and equine pericardium (78% and 67% versus 16% and 15%, respectively). The fraction of neutral *O*-glycans in equine pericardium (25%) was larger than in the other pericardial and valvular tissues (around 5-9% in all other tissues). The fraction of neutral *N*-glycans in the pericardial tissues was close to 30% (Fig. 3b and Table 2). Porcine aortic and pulmonary valve *N*-glycans had the lowest fraction of neutral structures (16%-17%), the largest fraction of Neu5Ac-sialylated *N*-glycans (82-83%), and the smallest fraction of Neu5Gc-containing *N*-glycans (≤12%). Porcine pulmonary epithelium has previously been reported to contain low (3%) levels of Neu5Gc-terminated *N*-glycans [32]. The fraction of Neu5Ac- and Neu5Gc-sialylated *N*-glycans in porcine and equine pericardium were similar, 66% and 14% respectively. *N*-glycans released from bovine pericardium had similar proportions of Neu5Ac- and Neu5Gc-sialylation (45% and 42%, respectively). Seven sialylated *O*-glycans were found to be sulfated (Table S1). MS/MS spectra of these structures revealed dominant Y ions (e.g. *m/z* 464 and 829 in Fig. 3c and d) suggesting loss of sialic acid and B/C ions (e.g. *m/z* 241 and 462 in Fig. 3c and d) suggesting sulfate-containing fragment ions.

### Distribution of the LacdiNAc determinant

**Fig. 4:**
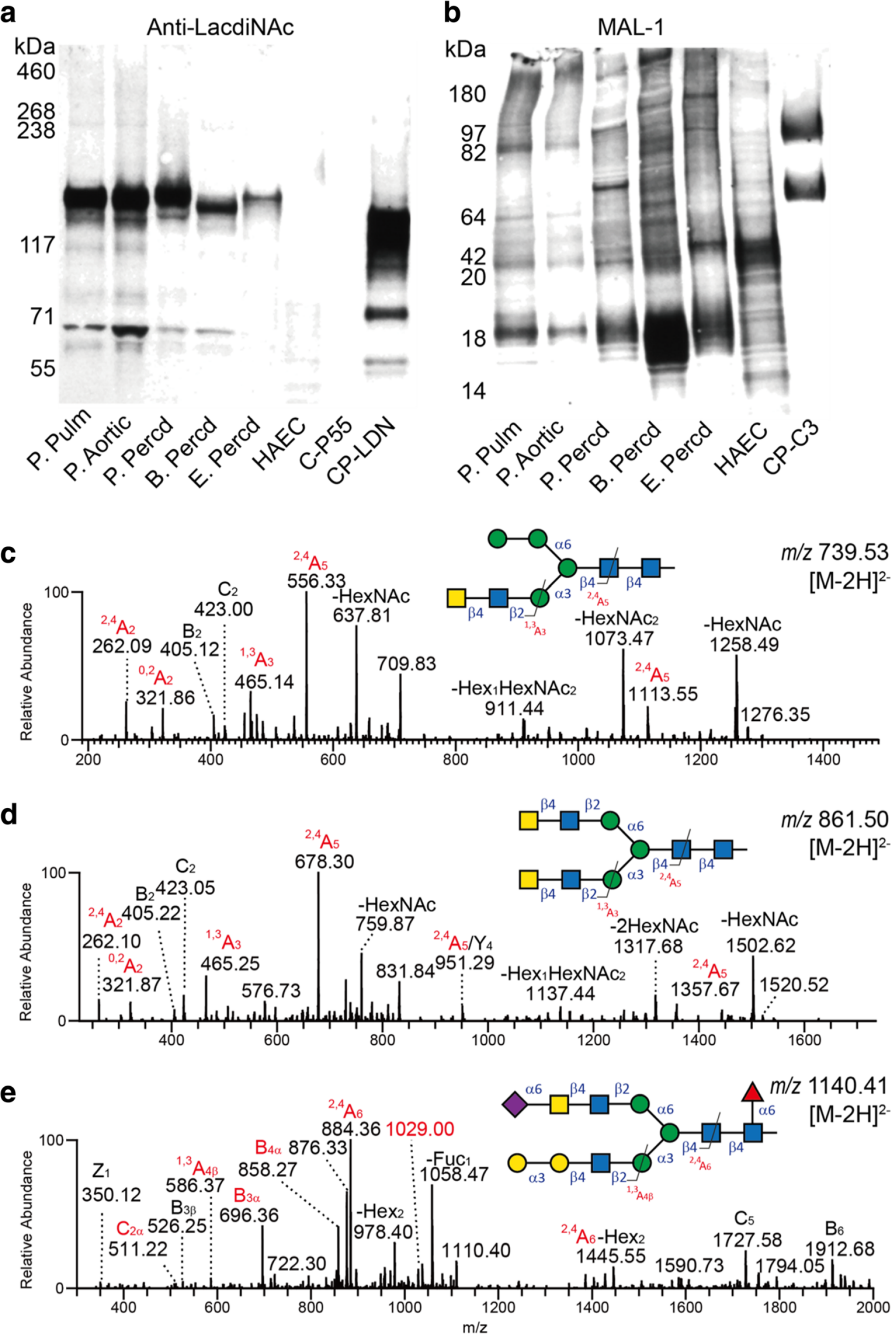
Western blot analysis of protein extracts from animal heart valves and pericardia using an anti-LacdiNAc antibody (**a**) and the MAL-1 lectin (**b**). A recombinant mucin-type fusion protein carrying terminal LacdiNAc determinants (CP-LDN) and purified from CHO-K1 cells transfected with plasmids encoding human B4GALNT3 and GCNT1 was used as a positive control. Positive control for MAL-1 staining was a mucin-type fusion protein carrying core 3 *O*-glycans extended with type 2 outer chains (CP-C3). A human aortic endothelial cell lysate was used as negative for LacdiNAc staining. MS/MS spectra of three LacdiNAc-containing *N*-glycans with masses corresponding to Hex_4_HexNAc_4_ ([M-2H]^2-^ of *m/z* 739), Hex_3_HexNAc_6_ ([M-2H]^2-^ of *m/z* 861), and Neu5Ac_1_Hex_5_HexNAc_5_deHex_1_ ([M-2H]^2-^ of *m/z* 1140) are shown in **c**, **d** and **e**, respectively

The anti-LacdiNAc antibody showed a clear reaction with a few glycoprotein species in the animal heart tissue lysates while only weakly stained components were found in the human aortic endothelial cellprotein lysate. The lectin, MAL-1, recognizes the type 2 chain (Galβ1,4GlcNAc or LacNAc) with or without α2,3-linked sialic acid. In contrast to the anti-LacdiNAc reactivity, MAL-1 stained multiple proteins in both the animal lysates as well as the human aortic endothelial celllysate. As for the MAL-2 and SNA staining (Fig. 3), the staining pattern of MAL-1 was similar in the porcine aortic and pulmonary valve tissues but varied between different porcine heart tissues and between the pericardia of different species (Fig. 4b).

The presence of sequences consistent with the LacdiNAc determinant was confirmed by LC-MS/MS (Fig. 4c-e) and found exclusively on *N*-glycans (10 structures identified). The presence of LacdiNAc was confirmed by the presence of fragment ions at *m/z* 405 and 423 (B_2_ and C_2_ ions) as well as cross-ring fragmentation giving rise to the ion at *m/z* 465 (^1,3^A_3_, Fig. 4c and d). Interestingly, an *N*-glycan with sialylated LacdiNAc was detected in porcine aortic valve tissue (Fig. 4e). In the MS^2^ spectrum of this structure (Fig. 4e), fragment ions at *m/z* 876 and 858 (C_4α_ and B_4α_) suggested a terminal Neu5Ac_1_Hex_1_HexNAc_2_ structure. The B_3α_ and C_2α_ ions at *m/z* 696 and 511 indicated that the terminal Neu5Ac was linked to the LacdiNAc determinant. The fragment ion at *m/z* 1029 ([M-2H]^2-^) suggests a loss of 221 Da from the parent ion. It was interpreted as a typical cross-ring (^0,2^X_Neu5Ac_) fragmentation of α2,6-linked Neu5Ac [33]. Thus, this structure was deduced to be a core fucosylated bi-antennary *N*-glycan with α-Gal on one branch and sialylated LacdiNAc with α2,6-linked Neu5Ac on the other branch. However, it is not clear whether sialylated LacdiNAc would react with the anti-LacdiNAc antibody or sialic acid-binding lectins such as SNA.

### Expression of Lewis blood group antigens

**Fig. 5:**
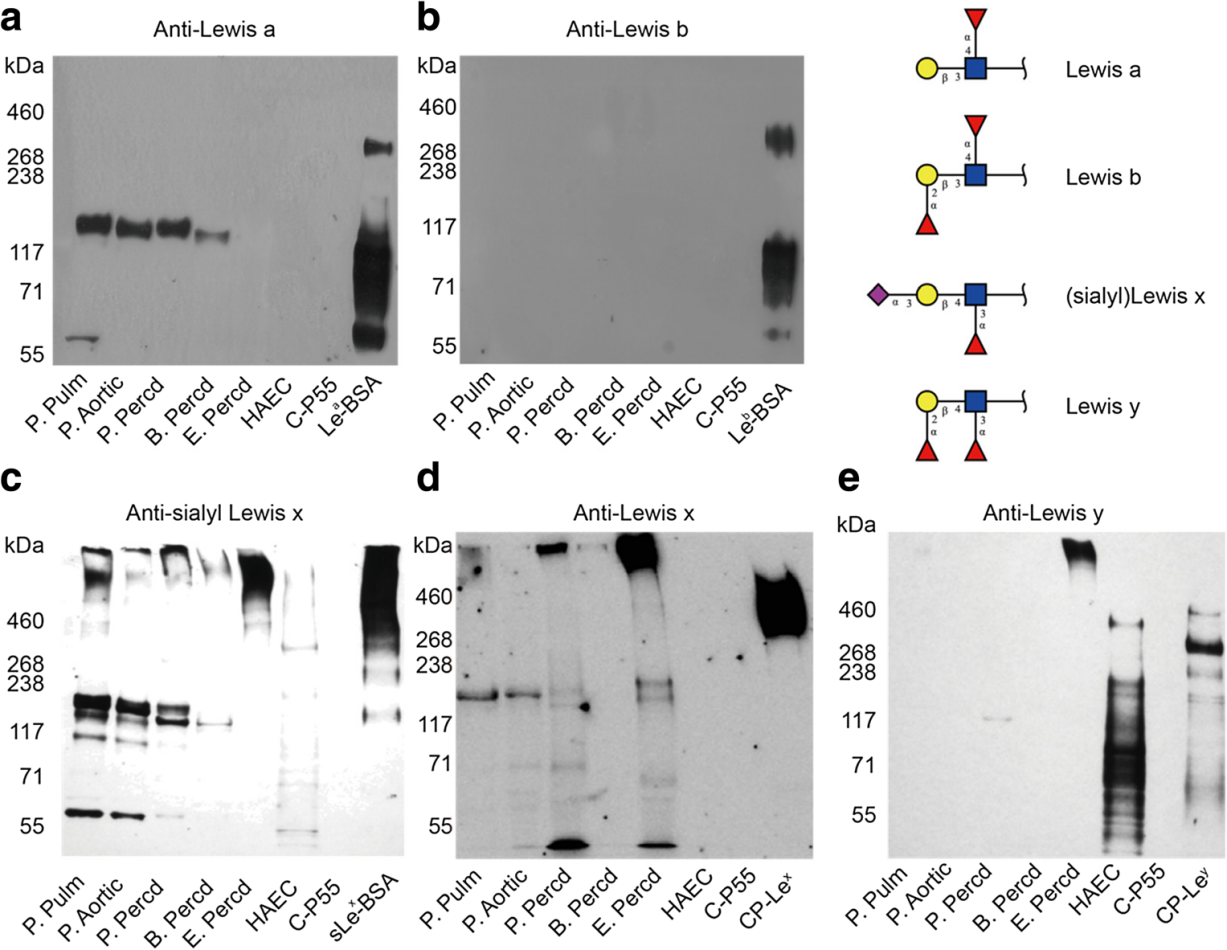
Western blot analysis of protein extracts from animal heart valves, pericardia and HAECs using anti-Le^a^ (**a**), anti-Le^b^ (**b**), anti-sialyl Le^x^ (**c**), anti-Le^x^ (**d**), and anti-Le^y^ (**e**) antibodies. Positive control samples were Le^a^-, Le^b^- and sLe^x^-BSA neoglycoconjugates. For Le^x^ and Le^y^ staining, recombinant mucin-type fusion proteins carrying Le^x^ (CP-Le^x^) or Le^y^ determinants (CP-Le^y^) were used as positive controls, while C-P55 was the negative control

The expression of blood group Lewis (Le^a^ and Le^b^) and similar antigens (Le^x^, Le^y^ and sLe^x^) on glycoproteins in the lysates of porcine, bovine and equine heart tissue was analyzed by Western blotting. Le^a^ determinants were detected in porcine and bovine heart tissue glycoproteins but not in equine pericardium and human aortic endothelial cells (Fig. 5a). No glycoproteins carrying Le^b^ determinants were detected in any of the tissues (Fig. 5b). sLe^x^ determinants were expressed on glycoproteins of all tissue protein lysates (Fig. 5c), while Le^x^ expression pattern was similar but weaker (Fig. 5d). Protein bands with an estimated molecular weight of approximately 55 kDa were exclusively stained with sLe^x^ (Fig. 5c). Le^y^ expression was restricted to components of equine pericardium and human aortic endothelial cells (Fig. 5e).

Only two *N*-glycans with terminal fucose, in addition to core fucosylation, were detected by LC-MS/MS. One was only detected in human aortic endothelial cells (*m/z* 2589 with a composition of NeuAc_1_Hex_6_HexNAc_5_deHex_2_, data not shown); while the other at *m/z* 2752 (NeuAc_1_Hex_7_HexNAc_5_deHex_2_) was detected in bovine and porcine pericardium (*m/z* 2752 in Table S1). This suggests that the overall presence of Le^a^/Le^x^-containing species were low in all samples. In addition, no sequences consistent with blood group AB(O)H antigens were detected in the protein lysates of animal heart tissues (Table S1).

### Expression of sulfated O-glycans

All sulfated glycans identified were found to be *O*-linked. One third of all *O*-glycans (14 out of 40) were sulfated, including two structures with two sulfate groups (Hex_2_HexNAc_2_Sul_2_ and Hex_3_HexNAc_3_Sul_2_ in Table S1). In contrast, sulfated *O*-glycans were not detected on glycoproteins in the HAEC lysate. Porcine, bovine and equine pericardia lysates had similar levels of sulfated *O*-glycans (9%, 12% and 11%, respectively, Table 2), while porcine pulmonary and aortic valve tissue had high levels of sulfated *O*-glycans (41 and 32%, respectively, Table 2).

## Discussion

It has been hypothesized that an immune response contributes to SVD [3]. Binding of immunoglobulins to antigens in the valve matrix, subsequent complement activation and recruitment of macrophages may be events triggering SVD [7, 8]. Identifying the targets for BHV-reactive antibodies may facilitate strategies to genetically alter the antigen expression, especially if it is of carbohydrate nature [34–37]. Most of the work done to characterize the repertoire of xenogeneic carbohydrate determinants such as α-Gal, Neu5Gc and others, on animal donor tissue for BHV has been performed using immunohistochemistry [8, 13, 18, 19]. Recognition of carbohydrate determinants by biological reagents is core chain-dependent [38–40]. Therefore, assessing antigen expression only by immunostaining has limitations. We used a combination of Western blotting and LC-MS/MS to characterize the *N*- and *O*-glycomes of protein lysates of porcine, bovine and equine heart tissues. Not only did this enable us to assess the representation of known xenogeneic determinants in the respective glycomes of the different tissues, but we could also identify which type of glycan and core chain carried a particular determinant and whether it was structurally modified. Since both α-Gal and Neu5Gc determinants have been identified in commercial valves used in the clinic (8, 25), it is a reasonable assumption that additional carbohydrate determinants identified on native tissues used for BHV manufacturing may be present following tissue processing, including glutaraldehyde fixation. It can also be anticipated that protein- compared to lipid-linked glycans are more likely to remain in the BHVs following processing.

In addition to the terminal α-Gal-determinant (generated by the *GGTA1* gene product), Neu5Gc (generated by the *CMAH* gene product), and the Sd^a^-like antigen (generated by the activity of the B4GALNT2 transferase) are believed to be immunogenic xenoantigens in humans and therefore may initiate an immune-mediated destruction of the BHVs [41, 42]. We showed that *N*- as well as *O*-glycans of porcine, bovine and equine heart tissues frequently carried terminal α-Gal and Neu5Gc determinants. The representation of α-Gal- and Neu5Gc-containing glycans varied between species. The highest representation of α-Gal- (36%) and Neu5Gc-containing (42%) glycans was found among the *N*-glycans of bovine pericardium. In contrast to the *N*-glycans, the relative amount of α-Gal-containing *O*-glycans was very low (<4%) in all pericardial tissues. Concordant with previous studies [32], the largest fraction of Neu5Gc-containing *O*-glycans was detected in porcine pericardium (65%). LC-MS/MS is a powerful method to establish tentative sequences of glycans carrying a certain determinant in a glycan mixture. However, to make a quantitative statement on absolute amounts of a particular glycan is more problematic. Structural diversity among glycans carrying xenogeneic determinants may not be the most important determinant of immunogenicity. Instead, factors such as the density of the xenogeneic determinant on the cell surface and the nature of the protein carrying the determinant may be just as important for the immunogenicity of the tissue.

We made two interesting observations regarding the repertoire of glycoproteins in the lysates carrying α-Gal (Fig. 2a and b). First, human IgM and IgG affinity-purified from human blood group AB serum pools on beads carrying the Galα1,3Gal-determinant exhibited distinct binding patterns on the lysates in Western blotting. Second, glycoprotein species carrying α-Gal appeared conserved between species and between tissues of the same species. In contrast, the binding pattern of sialic acid-specific lectins and anti-Neu5Gc antibodies on glycoproteins solubilized from the different pericardial tissues was different (Fig. 3a). Even though their binding pattern on proteins from porcine aortic and pulmonary valve tissue was similar, it was distinct from the reactivity on proteins from porcine pericardium (Fig. 3a). When a specific carbohydrate determinant, like for example α-Gal, is carried by a limited number of protein species that appears to be conserved between species and tissues, it is tempting to assume that it has a particular role to play on that very protein. However, it may only reflect a sub-compartmentalization of the secretory pathway such that a restricted number of proteins encounter the glycosyltransferases required to make the determinant in question.

There were no proteins in the lysates stained with the anti-Sd^a^ antibody (KM469; not shown) in any of the animal heart tissues even though this epitope has been detected in other tissues in pig, such as the large intestine [43]. The absence of Sd^a^ was also confirmed by LC-MS/MS. Instead, the LacdiNAc determinant was identified in all animal heart tissues by both Western blot and LC-MS/MS. The number of proteins carrying LacdiNAc was low based on Western blotting after SDS-PAGE and appeared conserved between species and tissues (Fig. 4a). LacdiNAc is the product of B4GALNT3 in man and mouse [44]. The B4GALNT3 analogues in other species have not been well characterized so far. Both LacdiNAc and Sd^a^, the latter one being the product of B4GALNT2, are recognized by the *Dolichos biflorus* agglutinin (DBA) [45]. Even though the expression levels of LacdiNAc on proteins solubilized from primary HAEC appeared to be low (Fig. 4a), the expression in composite human heart tissues is still unknown. LacdiNAc has been identified in human tissues [46]. Both LacdiNAc and Sd^a^ carry a terminal β1,4-linked GalNAc. Another potential xenoantigen, the Forssman (GalNAcα1,3GalNAcβ) antigen, which is carried by complex glycolipids and expressed in horse, sheep, mouse, hamster, chicken, and guinea pig, may be immunogenic in Forssman-negative species such as pigeon, rat, ox, rabbit and man [47–50]. Whether terminal GalNAc on *N*- and *O*-glycans in general are potential xenoantigens in man remains to be shown.

We also observed a high level (about 10% of the total amounts of *O*-glycans) of sulfated *O*-glycans on proteins solubilized from porcine, bovine and equine pericardium, and even higher levels on proteins from porcine pulmonary and aortic valve tissue (41 and 32%, respectively). Sulfated glycans are involved in many biological processes including cell adhesion, signaling, and growth factor presentation [51]. For example, sulfated and sialylated Lewis epitopes on *O*-glycans of high endothelial venules are ligands for L-selectin on lymphocytes essential for homing of lymphocytes to HEV [52, 53]. In a recent study, 330 different *N*- and *O*-glycans as well as glycopeptides and glycoproteins were screened for binding to IgG and IgM in serum from 135 healthy individuals. No increased binding activity to sulfated structures was detected [54]. However, only four sulfated structures were included in this array. It is unclear if the sulfated *O*-glycans we identified are experienced as non-self of the human immune system.

One *N*-glycan with sialylated LacdiNAc was detected in the porcine aortic valve tissue. Neu5Acα2,6GalNAcβ1,4GlcNAc has previously only been found on a limited number of peptide hormones such as the prolactin/growth hormone family members in pregnant rats [55] and in bovine milk glycoproteins such as lactoferrin [56]. Whether sialylated LacdiNAc is immunogenic in humans is not known.

In this study, glycan profiles of porcine, bovine and equine pericardium, and porcine pulmonary and aortic valve tissues were characterized to explore the distribution of known xenogeneic determinants such as α-Gal and Neu5Gc, but also to identify additional potential xenogeneic glycans. Identification of such potential xenogeneic glycans may allow future genetic engineering of livestock to prevent their expression rendering the donor tissue less immunogenic in humans.

## Electronic supplementary material

ESM 1 (DOCX 435 kb)

ESM 2 (XLSX 28 kb)

## Abbreviations

BHV: Bioprosthetic heart valves;
B. Percd: Bovine pericardium;
E. Percd: Equine pericardium
HAEC: Primary human aortic endothelial cell;
LacdiNAc: *N, N´*-diacetyllactosamine
MAL: *Maackia amurensis* lectin;
MHV: Mechanical heart valves;
P. Aortic: Porcine aortic valve;
P. Percd: Porcine pericardium;
P. Pulm: Porcine pulmonary valve;
Sd^a^: Sid blood group antigen;
SNA: *Sambucus nigra* lectin;
SVD: Structural valve deterioration
