# Supplementary material for: Identification by mass spectrometry and immunoblotting of xenogeneic antigens in the N- and O-glycomes of porcine, bovine and equine heart tissues
Source: Glycoconj J. 2020 Jun 15;37(4):485–98. doi: 10.1007/s10719-020-09931-1 (PMC7329767; doi:10.1007/s10719-020-09931-1)
Supplement: Supplementary file 1 — (DOCX 435 kb) [file 10719_2020_9931_MOESM1_ESM.docx]

**Identification of xenogeneic antigens in the *N*- and *O*-glycomes of porcine, bovine and equine heart tissues and their potential importance for bioprosthetic heart valve immunogenicity**

**Chunsheng Jin, Reeja Maria Cherian, Jining Liu, Heribert Playà, Cesare Galli, Niclas G. Karlsson, Michael E Breimer, and Jan Holgersson**

Table S1. List of proposed *N*- and *O*-glycan structures in the animal tissues identified by LC-MS/MS.

Table S2. MIRAGE

Table S3. URLs of LTQ-LC MS/MS raw data.

**Table S1. List of proposed *N*- and *O*-glycan structures in the animal tissues identified by LC-MS/MS.** The names of the structures are given in their reduced form in negative-ion mode ([M-H]-). The names of isomeric structures are separated by hyphens. The relative amounts of different structures are given in percentage (%) of the total sum of integrated peak areas in the LC-MS chromatograms. The relative percentage of *N*-glycans, *O*-glycans, and proteoglycans were calculated separately. A, glucuronic acid (
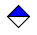
); B. Percd, bovine pericardium; E. Percd, equine pericardium; F, fucose (
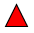
); H, hexose standing for either mannose (
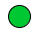
), galactose (
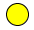
) or glucose (
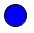
); N, *N*-acetylhexosamine standing for either *N*-acetylglucosamine (
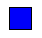
) or *N*-acetylgalactosamine (
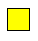
); Na, *N*-acetylneuraminic acid (
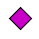
); Ng, *N*-glycolylneraminic acid (
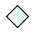
); P. Aortic, porcine aorta; P. Percd, porcine pericardium; P. Pulm, porcine pulmonary; S, sulfate; X, xylose (
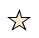
).

| **Name** | **Composition** | **Putative structures** | **E. Percd** | **B. Percd** | **P. Percd** | **P. Pulm** | **P. Aortic** |
| --- | --- | --- | --- | --- | --- | --- | --- |
| 425 | N_2_ | _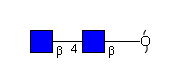_ | 0.8 | 0.0 | 0.0 | 0.0 | 0.0 |
| 571 | N_2_F_1_ | _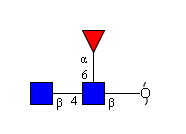_ | 0.0 | 0.3 | 0.2 | 0.1 | 0.1 |
| 749 | H_2_N_2_ | _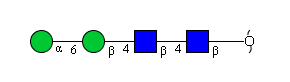_ | 0.0 | 0.1 | 0.2 | 0.1 | 0.1 |
| 895 | H_2_N_2_F_1_ | _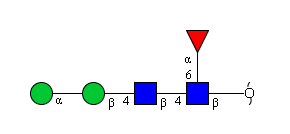_ | 0.0 | 0.1 | 0.0 | 0.0 | 0.0 |
| 1114 | H_3_N_3_ | _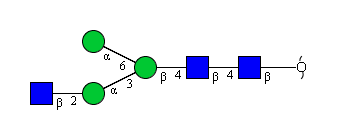_ | 0.0 | 0.0 | 0.2 | 0.1 | 0.1 |
| 1235 | H_5_N_2_ | _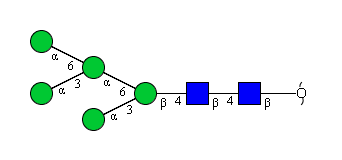_ | 2.9 | 1.7 | 1.1 | 0.6 | 0.6 |
| 1260 | H_3_N_3_F_1_ | _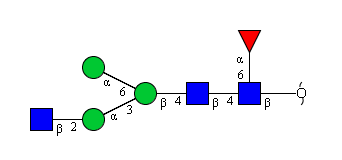_ | 0.0 | 0.0 | 0.0 | 0.1 | 0.1 |
| 1276 | H_4_N_3_ | _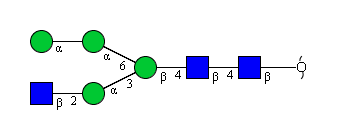_ | 0.2 | 0.0 | 0.5 | 0.0 | 0.0 |
| 1317-1 | H_3_N_4_ | _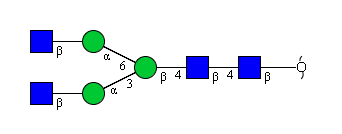_ | 0.0 | 0.0 | 0.0 | 0.1 | 0.1 |
| 1317-2 | H_3_N_4_ | _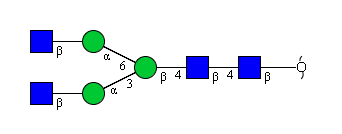_ | 0.3 | 0.1 | 0.0 | 1.4 | 1.5 |
| 1397 | H_6_N_2_ | _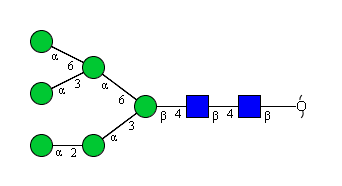_ | 2.2 | 2.2 | 1.1 | 1.0 | 1.1 |
| 1438-1 | H_5_N_3_ | _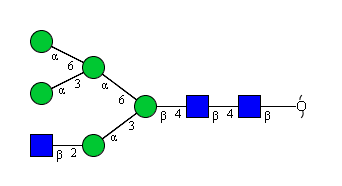_ | 0.2 | 0.0 | 0.0 | 0.0 | 0.0 |
| 1438-2 | H_5_N_3_ | _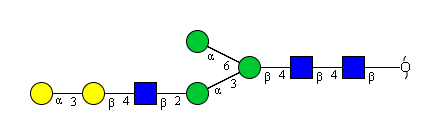_ | 0.0 | 0.1 | 0.0 | 0.0 | 0.0 |
| 1463 | H_3_N_4_F_1_ | _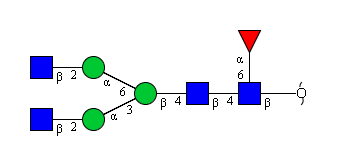_ | 2.5 | 0.7 | 3.2 | 3.5 | 3.7 |
| 1479-1 | H_4_N_4_ | _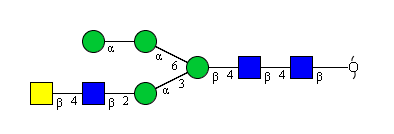_ | 0.0 | 0.0 | 0.0 | 0.2 | 0.2 |
| 1479-2 | H_4_N_4_ | _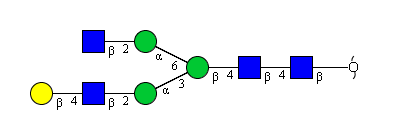_ | 0.1 | 0.0 | 0.0 | 0.0 | 0.0 |
| 1479-3 | H_4_N_4_ | _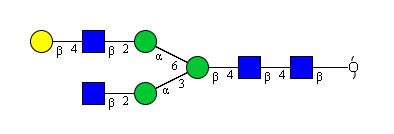_ | 0.0 | 0.3 | 0.7 | 0.7 | 0.8 |
| 1520 | H_3_N_5_ | _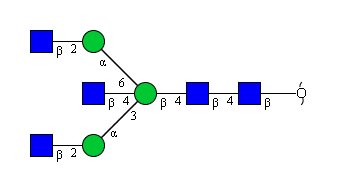_ | 0.0 | 0.3 | 0.1 | 0.0 | 0.0 |
| 1559-1 | H_7_N_2_ | _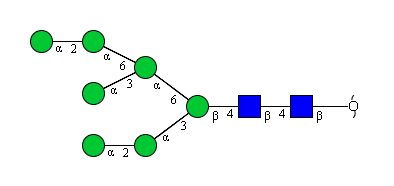_ | 0.5 | 0.8 | 0.2 | 0.2 | 0.2 |
| 1559-2 | H_7_N_2_ | _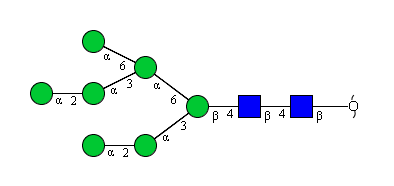_ | 0.0 | 0.0 | 0.2 | 0.2 | 0.3 |
| 1600-1 | H_6_N_3_ | _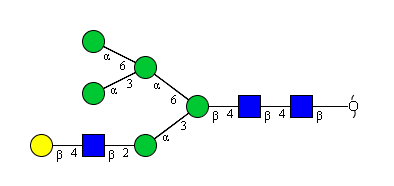_ | 0.8 | 0.4 | 0.2 | 0.1 | 0.1 |
| 1600-2 | H_6_N_3_ | _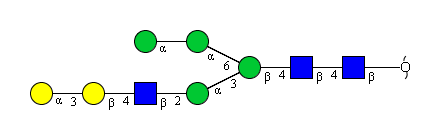_ | 0.4 | 0.1 | 0.2 | 0.1 | 0.0 |
| 1625-1 | H_4_N_4_F_1_ | _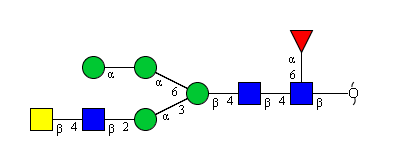_ | 0.0 | 0.0 | 0.1 | 0.3 | 0.3 |
| 1625-2 | H_4_N_4_F_1_ | _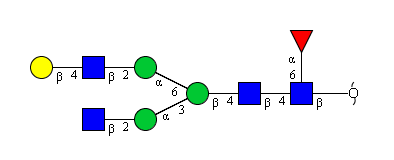_ | 1.4 | 1.9 | 1.6 | 1.4 | 1.4 |
| 1641-1 | H_5_N_4_ | _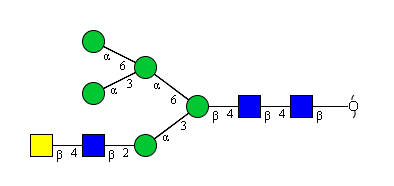_ | 0.1 | 0.0 | 0.0 | 0.1 | 0.0 |
| 1641-2 | H_5_N_4_ | _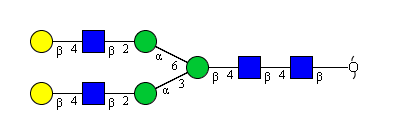_ | 0.2 | 0.6 | 0.2 | 0.1 | 0.1 |
| 1641-3 | H_5_N_4_ | _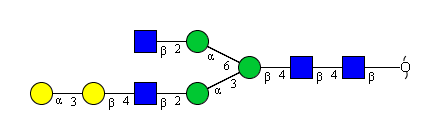_ | 0.0 | 0.0 | 0.1 | 0.0 | 0.0 |
| 1666-1 | H_3_N_5_F_1_ | _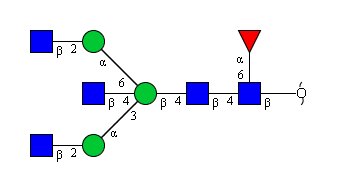_ | 0.0 | 0.4 | 0.2 | 0.0 | 0.0 |
| 1666-2 | H_3_N_5_F_1_ | _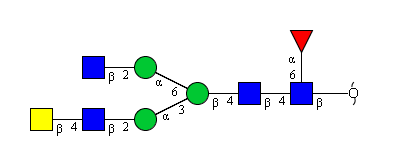_ | 0.0 | 0.1 | 0.0 | 0.2 | 0.1 |
| 1682 | H_4_N_5_ | _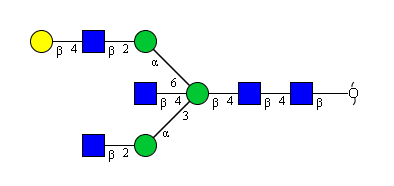_ | 0.0 | 3.5 | 0.6 | 0.0 | 0.0 |
| 1721-1 | H_8_N_2_ | _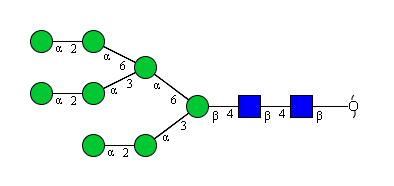_ | 0.8 | 0.0 | 0.0 | 0.0 | 0.0 |
| 1721-2 | H_8_N_2_ | _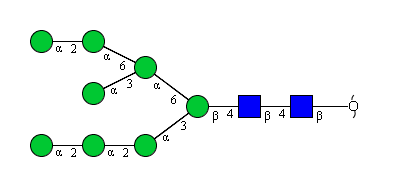_ | 0.0 | 1.1 | 0.8 | 0.6 | 0.7 |
| 1723 | H_3_N_6_ | _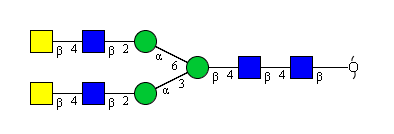_ | 0.0 | 0.0 | 0.0 | 0.0 | 0.1 |
| 1729-1 | Na_1_H_5_N_3_ | _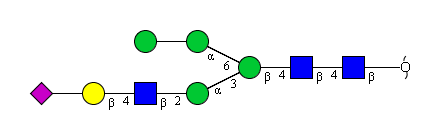_ | 0.2 | 0.0 | 0.0 | 0.0 | 0.0 |
| 1729-2 | Na_1_H_5_N_3_ | _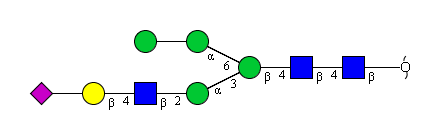_ | 0.4 | 0.0 | 0.0 | 0.0 | 0.0 |
| 1746 | H_6_N_3_F_1_ | _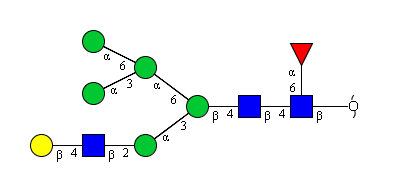_ | 0.0 | 0.0 | 0.2 | 0.0 | 0.0 |
| 1762-1 | H_7_N_3_ | _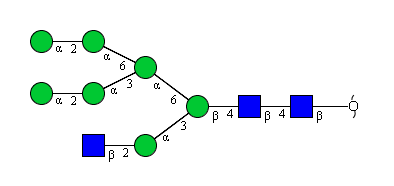_ | 0.1 | 0.0 | 0.0 | 0.0 | 0.0 |
| 1762-1 | H_7_N_3_ | _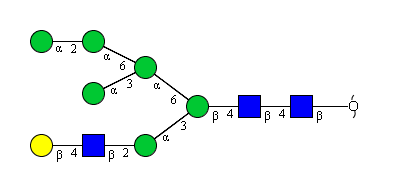_ | 0.0 | 0.1 | 0.0 | 0.0 | 0.0 |
| 1762-3 | H_7_N_3_ | _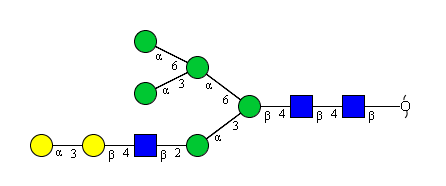_ | 0.9 | 0.2 | 0.4 | 0.0 | 0.0 |
| 1770 | Na_1_H_4_N_4_ | _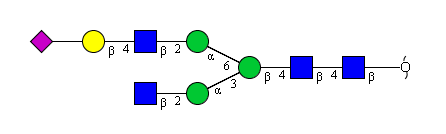_ | 0.2 | 0.0 | 0.0 | 0.0 | 0.0 |
| 1787-1 | H_5_N_4_F_1_ | _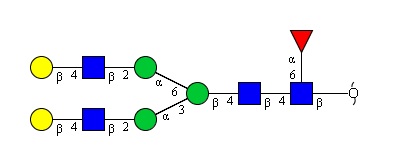_ | 1.1 | 2.7 | 0.7 | 0.2 | 0.3 |
| 1787-2 | H_5_N_4_F_1_ | _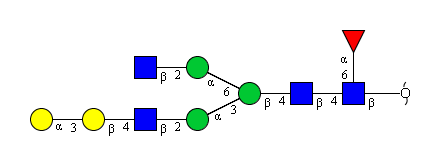_ | 0.9 | 0.4 | 0.9 | 0.6 | 0.0 |
| 1803 | H_6_N_4_ | _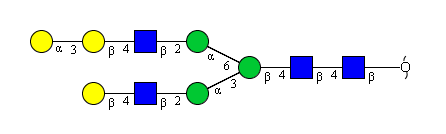_ | 0.5 | 0.5 | 0.4 | 0.0 | 0.0 |
| 1828 | H_4_N_5_F_1_ | _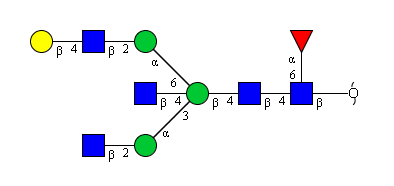_ | 0.0 | 0.5 | 0.1 | 0.0 | 0.0 |
| 1844-1 | H_5_N_5_ | _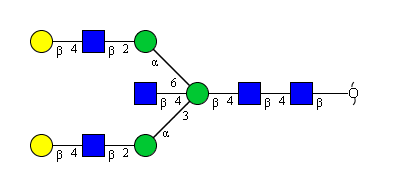_ | 0.0 | 0.3 | 0.0 | 0.0 | 0.0 |
| 1844-2 | H_5_N_5_ | _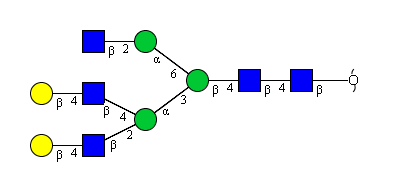_ | 0.0 | 0.0 | 0.2 | 0.0 | 0.0 |
| 1869-1 | H_3_N_6_F_1_ | _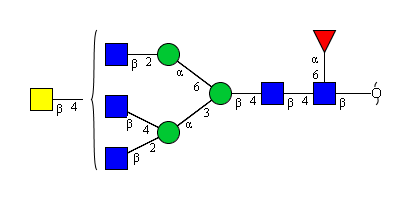_ | 0.0 | 0.0 | 0.0 | 0.0* | 0.0 |
| 1869-2 | H_3_N_6_F_1_ | _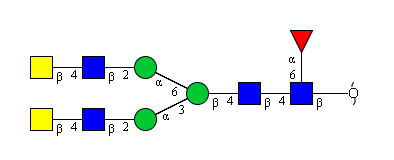_ | 0.4 | 0.0 | 0.0 | 0.8 | 0.8 |
| 1883 | H_9_N_2_ | _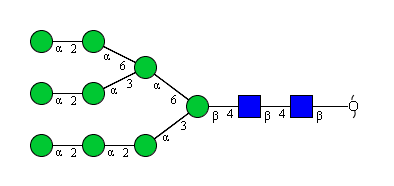_ | 1.4 | 0.8 | 1.1 | 0.7 | 0.8 |
| 1932-1 | Na_1_H_5_N_4_ | _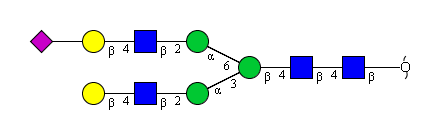_ | 2.4 | 0.2 | 0.4 | 1.3 | 1.4 |
| 1932-2 | Na_1_H_5_N_4_ | _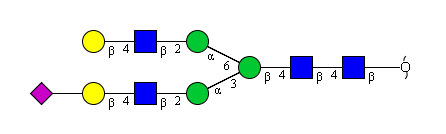_ | 0.0 | 0.7 | 0.0 | 0.0 | 0.0 |
| 1933 | Ng_1_H_4_N_4_F_1_ | _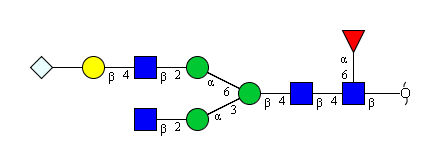_ | 0.0 | 0.0 | 0.6 | 0.4 | 0.0 |
| 1949 | H_6_N_4_F_1_ | _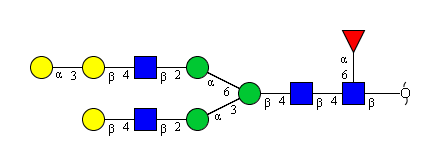_ | 2.3 | 2.5 | 1.4 | 0.8 | 0.7 |
| 1965 | H_7_N_4_ | _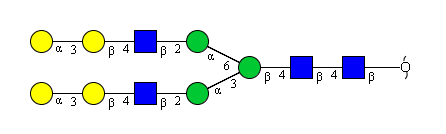_ | 1.0 | 0.7 | 0.7 | 0.0 | 0.0 |
| 1990-1 | H_5_N_5_F_1_ | _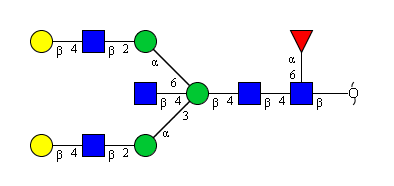_ | 0.0 | 0.2 | 0.2 | 0.0 | 0.0 |
| 1990-2 | H_5_N_5_F_1_ | _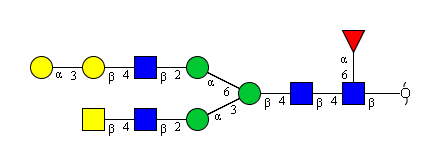_ | 1.1 | 0.2 | 0.1 | 0.7 | 0.7 |
| 2045 | H_10_N_2_ | _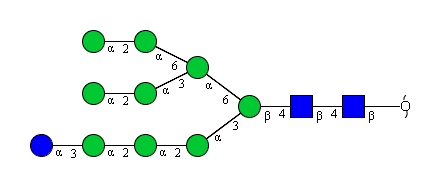_ | 0.1 | 0.0 | 0.0 | 0.0 | 0.0 |
| 2078-1 | Na_1_H_5_N_4_F_1_ | _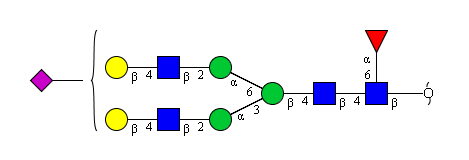_ | 0.0 | 0.2 | 1.7 | 2.6 | 2.6 |
| 2078-2 | Na_1_H_5_N_4_F_1_ | _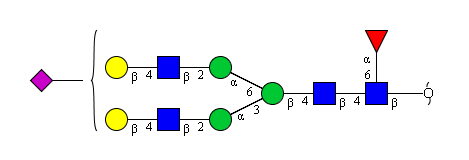_ | 1.9 | 0.0 | 0.6 | 0.6 | 0.0 |
| 2078-3 | Na_1_H_5_N_4_F_1_ | _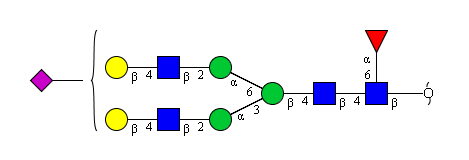_ | 0.0 | 1.9 | 0.0 | 0.0 | 0.4 |
| 2094-1 | Ng_1_H_5_N_4_F_1_ | _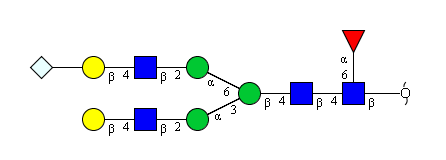_ | 0.0 | 0.6 | 0.6 | 0.4 | 0.5 |
| 2094-2 | Ng_1_H_5_N_4_F_1_ | _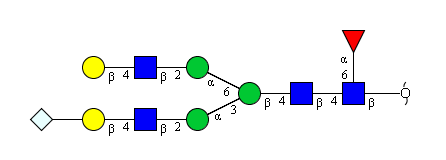_ | 0.8 | 1.7 | 0.0 | 0.0 | 0.0 |
| 2110 | Ng_1_H_6_N_4_ | _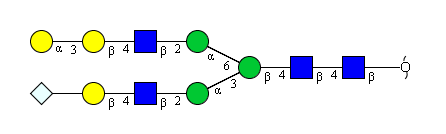_ | 3.3 | 0.5 | 1.4 | 0.0 | 0.0 |
| 2111 | H_7_N_4_F_1_ | _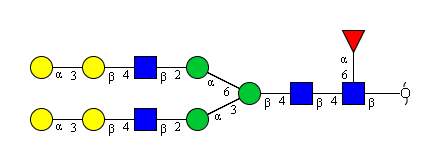_ | 4.5 | 5.3 | 5.1 | 0.1 | 1.5 |
| 2152 | H_6_N_5_F_1_ | _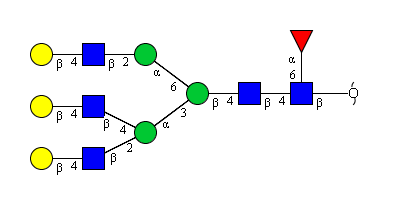_ | 0.0 | 0.1 | 0.1 | 0.0 | 0.0 |
| 2223-1 | Na_2_H_5_N_4_ | _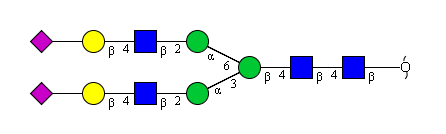_ | 37.8 | 0.0 | 0.0 | 0.0 | 0.0 |
| 2223-2 | Na_2_H_5_N_4_ | _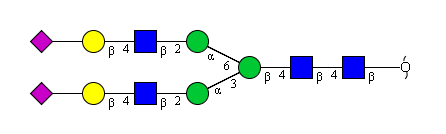_ | 0.0 | 0.9 | 3.8 | 4.9 | 5.3 |
| 2223-3 | Na_2_H_5_N_4_ | _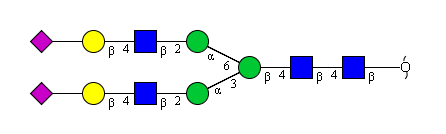_ | 0.0 | 0.0 | 1.4 | 2.0 | 1.8 |
| 2239-1 | Na_1_Ng_1_H_5_N_4_ | _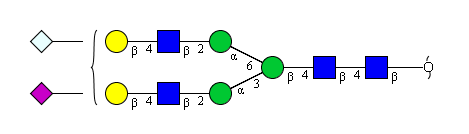_ | 3.8 | 0.0 | 0.0 | 0.0 | 0.0 |
| 2239-2 | Na_1_Ng_1_H_5_N_4_ | _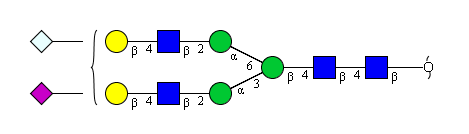_ | 0.0 | 1.6 | 0.3 | 0.4 | 0.4 |
| 2240-1 | Na_1_H_6_N_4_F_1_ | _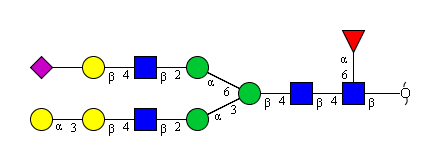_ | 1.9 | 0.0 | 0.0 | 0.0 | 0.0 |
| 2240-2 | Na_1_H_6_N_4_F_1_ | _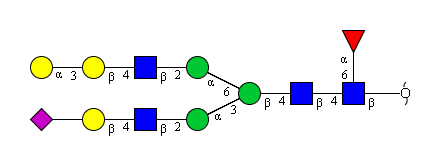_ | 3.7 | 8.1 | 1.3 | 2.5 | 1.9 |
| 2256-1 | Ng_1_H_6_N_4_F_1_ | _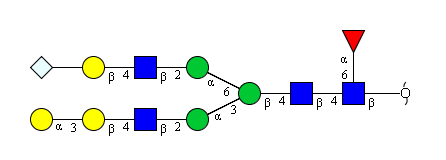_ | 1.4 | 8.3 | 1.1 | 0.7 | 0.7 |
| 2256-2 | Ng_1_H_6_N_4_F_1_ | _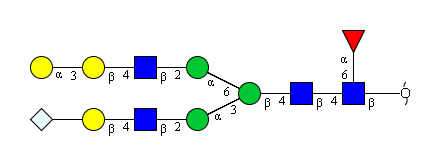_ | 1.2 | 5.4 | 2.9 | 0.3 | 0.0 |
| 2281-1 | Na_1_H_5_N_5_F_1_ | _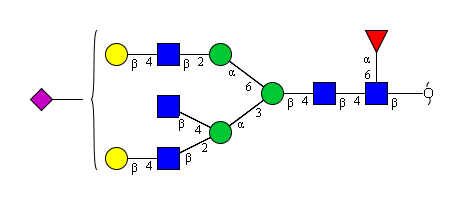_ | 0.0 | 0.8 | 0.0 | 0.0 | 0.0 |
| 2281-2 | Na_1_H_5_N_5_F_1_ | _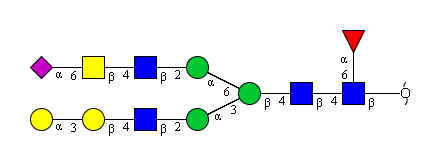_ | 0.0 | 0.0 | 0.0 | 0.0 | 0.1 |
| 2338 | Na_1_H_5_N_6_ | _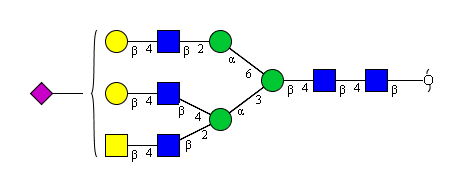_ | 0.0 | 0.0 | 1.0 | 0.8 | 0.0 |
| 2369-1 | Na_2_H_5_N_4_F_1_ | _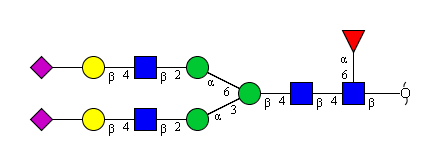_ | 3.7 | 0.0 | 0.0 | 0.0 | 0.0 |
| 2369-2 | Na_2_H_5_N_4_F_1_ | _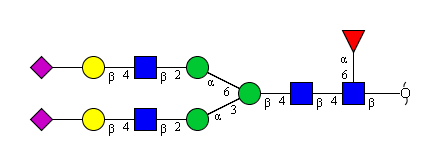_ | 0.0 | 0.8 | 47.1 | 58.5 | 56.0 |
| 2369-4 | Na_2_H_5_N_4_F_1_ | _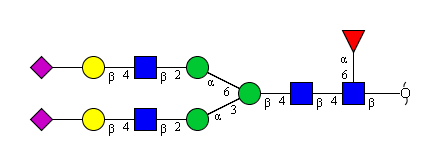_ | 4.3 | 8.9 | 0.0 | 0.0 | 0.0 |
| 2385-1 | Na_1_Ng_1_H_5_N_4_F_1_ | _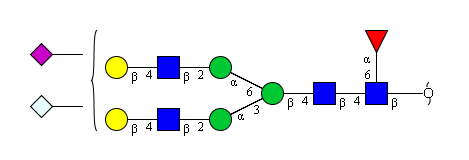_ | 0.0 | 0.0 | 7.6 | 7.7 | 10.5 |
| 2385-2 | Na_1_Ng_1_H_5_N_4_F_1_ | _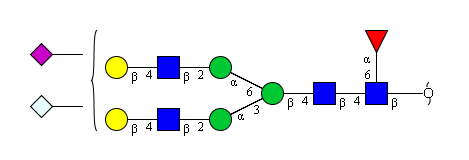_ | 1.3 | 1.0 | 0.0 | 0.0 | 0.0 |
| 2385-3 | Na_1_Ng_1_H_5_N_4_F_1_ | _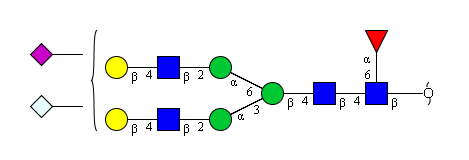_ | 0.6 | 7.9 | 0.0 | 0.0 | 0.0 |
| 2401-1 | Ng_2_H_5_N_4_F_1_ | _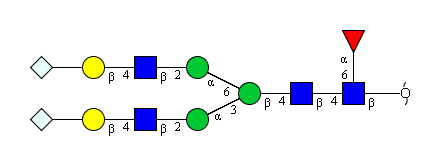_ | 0.6 | 0.4 | 0.8 | 0.0 | 0.0 |
| 2401-2 | Ng_2_H_5_N_4_F_1_ | _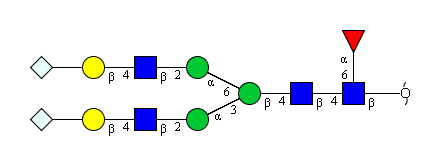_ | 0.0 | 2.5 | 2.6 | 0.0 | 0.0 |
| 2531 | Na_2_Ng_1_H_5_N_4_ | _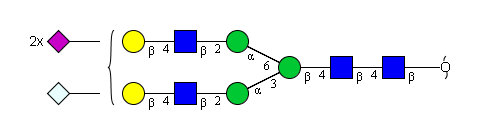_ | 0.0 | 3.9 | 0.0 | 0.0 | 0.0 |
| 2547 | Na_1_Ng_2_H_5_N_4_ | _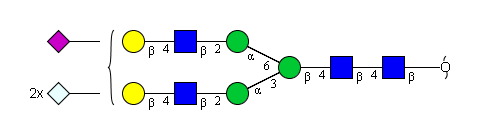_ | 0.0 | 6.4 | 0.0 | 0.0 | 0.0 |
| 2605 | Na_1_H_7_N_5_F_1_ | _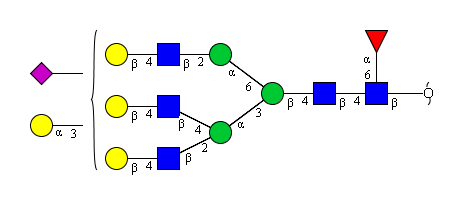_ | 0.7 | 1.2 | 0.0 | 0.0 | 0.0 |
| 2734-1 | Na_2_H_6_N_5_F_1_ | _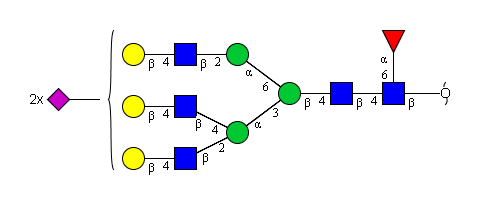_ | 0.6 | 0.0 | 0.0 | 0.0 | 0.0 |
| 2734-2 | Na_2_H_6_N_5_F_1_ | _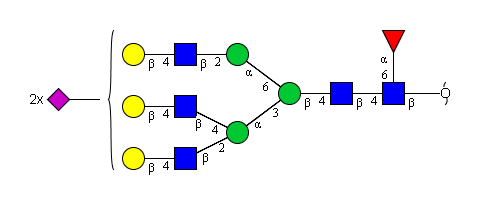_ | 0.0 | 0.2 | 0.0 | 0.6 | 0.0 |
| 2752 | Na_1_H_7_N_5_F_2_ |  | 0.0 | 2.5 | 0.3 | 0.0 | 0.0 |
| 2896 | Na_2_Ng_1_H_6_N_5_ |  | 0.5 | 0.0 | 0.0 | 0.0 | 0.0 |
| 2970 | Na_1_Ng_1_H_7_N_6_ |  | 0.0 | 0.4 | 0.0 | 0.0 | 0.0 |
| 3026 | Na_3_H_6_N_5_F_1_ |  | 0.0 | 0.0 | 1.4 | 1.4 | 2.0 |
| 3042 | Na_2_Ng_1_H_6_N_5_F_1_ |  | 0.2 | 0.0 | 0.0 | 0.0 | 0.0 |
| 3100 | Na_2_H_7_N_6_F_1_ |  | 0.0 | 1.5 | 0.0 | 0.0 | 0.0 |
| 3116 | Na_1_Ng_1_H_7_N_6_F_1_ |  | 0.3 | 1.5 | 0.0 | 0.0 | 0.0 |
| 3336 | Na_1_H_9_N_7_F_1_ |  | 0.2 | 0.0 | 0.0 | 0.0 | 0.0 |
| 3465 | Na_2_H_8_N_7_F_1_ |  | 0.4 | 0.5 | 0.0 | 0.0 | 0.0 |
| 3830 | Na_2_H_9_N_8_F_1_ |  | 0.1 | 0.0 | 0.0 | 0.0 | 0.0 |
| 384 | H_1_N_1_ |  | 18.6 | 1.8 | 1.0 | 0.6 | 0.5 |
| 425 | N_2_ |  | 1.4 | 0.2 | 0.0 | 0.2 | 0.1 |
| 464-1 | H_1_N_1_S_1_ |  | 4.9 | 0.0 | 0.0 | 0.0 | 0.0 |
| 464-2 | H_1_N_1_S_1_ |  | 0.0 | 6.4 | 4.5 | 0.6 | 0.3 |
| 667 | H_1_N_2_S_1_ |  | 0.0 | 0.0 | 0.0 | 0.5 | 0.2 |
| 675-1 | Na_1_H_1_N_1_ |  | 4.3 | 2.8 | 2.7 | 5.3 | 3.5 |
| 675-2 | Na_1_H_1_N_1_ |  | 25.8 | 30.4 | 10.5 | 13.8 | 14.2 |
| 691-1 | Ng_1_H_1_N_1_ |  | 1.3 | 1.0 | 5.7 | 0.5 | 0.4 |
| 691-2 | Ng_1_H_1_N_1_ |  | 4.0 | 5.8 | 9.5 | 0.9 | 0.8 |
| 749 | H_2_N_2_ |  | 0.0 | 0.3 | 0.0 | 0.8 | 0.3 |
| 755 | Na_1_H_1_N_1_S_1_ |  | 5.2 | 4.1 | 1.3 | 1.1 | 1.0 |
| 771 | Ng_1_H_1_N_1_S_1_ |  | 1.2 | 1.4 | 2.7 | 0.1 | 0.0 |
| 829 | H_2_N_2_S_1_ |  | 0.0 | 0.0 | 0.0 | 4.5 | 1.2 |
| 894 | Ng_1_H_1_N_2_ |  | 0.0 | 0.0 | 5.5 | 0.0 | 0.0 |
| 909 | H_2_N_2_S_2_ |  | 0.0 | 0.0 | 0.0 | 0.6 | 0.0 |
| 911-1 | H_3_N_2_ |  | 0.0 | 0.4 | 1.0 | 0.5 | 0.4 |
| 911-2 | H_3_N_2_ |  | 0.0 | 0.1 | 0.2 | 0.0 | 0.0 |
| 966 | Na_2_H_1_N_1_ |  | 24.7 | 27.8 | 11.1 | 18.9 | 25.7 |
| 982-1 | Na_1_Ng_1_H_1_N_1_ |  | 0.0 | 1.4 | 0.0 | 0.0 | 0.0 |
| 982-2 | Na_1_Ng_1_H_1_N_1_ |  | 7.1 | 0.0 | 11.5 | 2.4 | 3.2 |
| 991 | H_3_N_2_S_1_ |  | 0.0 | 0.0 | 0.0 | 0.9 | 1.0 |
| 998 | Ng_2_H_1_N_1_ |  | 1.6 | 2.9 | 10.4 | 0.7 | 0.0 |
| 1040-1 | Na_1_H_2_N_2_ |  | 0.0 | 0.3 | 0.0 | 0.4 | 0.3 |
| 1040-2 | Na_1_H_2_N_2_ |  | 0.0 | 1.3 | 0.0 | 5.1 | 6.4 |
| 1056 | Ng_1_H_2_N_2_ |  | 0.0 | 0.0 | 2.5 | 0.7 | 0.8 |
| 1120-1 | Na_1_H_2_N_2_S_1_ |  | 0.0 | 0.0 | 0.2 | 2.0 | 1.5 |
| 1120-2 | Na_1_H_2_N_2_S_1_ |  | 0.0 | 0.0 | 0.0 | 28.1 | 25.8 |
| 1136 | Ng_1_H_2_N_2_S_1_ |  | 0.0 | 0.0 | 0.0 | 1.7 | 0.0 |
| 1202 | Na_1_H_3_N_2_ |  | 0.0 | 0.9 | 0.4 | 0.4 | 0.6 |
| 1218 | Ng_1_H_3_N_2_ |  | 0.0 | 0.6 | 1.8 | 0.1 | 0.0 |
| 1259 | Ng_1_H_2_N_3_ |  | 0.0 | 0.0 | 0.0 | 0.1 | 0.0 |
| 1274 | H_3_N_3_S_2_ |  | 0.0 | 0.0 | 0.0 | 0.2 | 0.0 |
| 1282 | Na_1_H_3_N_2_S_1_ |  | 0.0 | 0.0 | 0.0 | 0.3 | 0.0 |
| 1331 | Na_2_H_2_N_2_ |  | 0.0 | 6.5 | 2.3 | 6.8 | 8.8 |
| 1347 | Na_1_Ng_1_H_2_N_2_ |  | 0.0 | 1.6 | 4.4 | 0.0 | 1.2 |
| 1363 | Ng_2_H_2_N_2_ |  | 0.0 | 0.7 | 4.3 | 0.4 | 0.5 |
| 1405 | Na_1_H_3_N_3_ |  | 0.0 | 0.0 | 0.0 | 0.2 | 0.2 |
| 1485 | Na_1_H_3_N_3_S_1_ |  | 0.0 | 0.0 | 0.0 | 0.4 | 0.6 |
| 1696 | Na_2_H_3_N_3_ |  | 0.0 | 1.1 | 0.0 | 0.4 | 0.6 |
| 1728 | Ng_2_H_3_N_3_ |  | 0.0 | 0.0 | 6.7 | 0.0 | 0.0 |
| 651 | H_2_A_1_X_1_ |  | 0.0 | 0.0 | 0.0 | 38.6 | 0.0 |
| 854 | H_2_N_1_A_1_X_1_ |  | 0.0 | 0.0 | 0.0 | 61.4 | 100.0 |
